# Screening and genomic identification of the bacterial pathogens from an apple orchard in Kazakhstan

**DOI:** 10.7717/peerj.21078

**Published:** 2026-06-23

**Authors:** Valeriya Kostyukova, Alexandr Pozharskiy, Gulnaz Nizamdinova, Ainur Khalilayeva, Aisha Taskuzhina, Dilyara Gritsenko

**Affiliations:** 1 Labratory of Molecular Biology, Institute of Plant Biology and Biotechnology, Almaty, Kazakhstan; 2 Research Center AgriBioTech, Almaty, Kazakhstan; 3 Department of Molecular Biology and Genetics, Al Farabi Kazakh National University, Almaty, Kazakhstan

**Keywords:** *Erwinia amylovora*, *Pseudomonas syringae*, Whole genome sequencing, *Malus domestica*

## Abstract

Apple production in Kazakhstan faces increasing threats from bacterial pathogens, including *Erwinia amylovora*, the causal agent of fire blight, and members of the *Pseudomonas syringae* species complex. These diseases not only cause substantial economic losses but also endanger the endemic wild apple *Malus sieversii*. In this study, apple leaves displaying symptoms of a bacterial infection were collected near Almaty and analyzed using molecular and genomic approaches. The preliminary low coverage genome sequencing and targeted 16S amplicon sequencing indicated the presence of *E. amylovora* and *Pseudomonas* spp. Subsequently, whole-genome sequencing (WGS) was performed on two *Erwinia amylovora* isolates and three *Pseudomonas* spp. isolates for definitive identification and phylogenetic placemenet. Two isolates were confirmed as *E. amylovora*, closely related to widely prevalent (WP) group, while three isolates belonged to *P. syringae* species group and included *P. fragariae*, *P. syringae*, and *P. tremae*. The presence of *P. tremae* and *P. fragariae* was reported for the first time in Kazakhstan. The results highlight the challenges of differentiating phenotypically similar phytopathogens and the need for the correct choice of diagnostic approach. Immunological tests and species-specific polymerase chain reaction (PCR) allow reliable detection and discrimination of *E. amylovora*, whereas genomic methods are particularly important for accurate taxonomic resolution of closely related isolates and for assessing the genetic variability of local populations. These data expand the genomic resources on plant pathogens in Central Asia and provide a foundation for management strategies by enabling more accurate pathogen identification and confirming the composition of regional *Pseudomonas* populations, thereby improving the interpretation of monitoring results and the selection of appropriate control measures.

## Introduction

The spread of pathogens, particularly bacterial diseases, of apple trees is an important problem in Kazakhstan as they not only cause significant economic losses by affecting yields of *Malus domestica,* as well as other fruit tree crops, but also threaten the populations of the wild Sievers’s apple (*Malus sieversii*), a rare endemic species of Tian-Shan mountain range. The efficient disease monitoring and control relies on the development of accurate and rapid diagnostic methods (Forsline, Luby & Aldwinckle, 2008; Harshman et al., 2017) which, in turn, depend on the availability of the genomic data. Apple trees may be susceptible to a variety of bacterial diseases, including blister spot and blister blight caused by *Pseudomonas syringae*, crown gall and hairy root caused by *Agrobacterium* spp., and, the most common and dangerous diseases, fire blight caused by *Erwinia amylovora* (Grove et al., 2003). *Erwinia amylovora*, is a globally distributed pathogen of fruit tree crops (Ke et al., 2024) that causes significant damage to agriculture. In China, the countries of the European Union, and according to EPPO classification, it is included in the list of quarantine organisms (EPPO, 2022). Its timely detection determines the effectiveness of control measures and reduces the risk of excessive applications of antibiotics (Singh et al., 2021). In China, fire blight has previously been reported to cause a 30–50% reduction in pear yields (Zeng, Slack & Amine Hassani, 2023). Notably, due to the high level of cross-border trade and geographical proximity, Chinese isolates of *E. amylovora* are closely related to isolates from Kyrgyzstan and Kazakhstan, confirming the spread and circulation of the pathogen in the Asian region (Wang et al., 2025). In Kazakhstan, the disease was first reported in 2008. In the 2010s, up to 50–60% of apple (*M. domestica*) and pear (*Pyrus communis*) trees were severely affected on certain farms (Maltseva et al., 2025).

Another bacterial pathogen widely affecting apple trees is *Pseudomonas syringae*, which infects leaves, buds, and bark, and also provokes the development of “blister spot” on fruits (Kennelly et al., 2007). The most typical symptoms of infection include necrosis of dormant buds, leaf spotting and blackening, discoloration of veins and petioles, dieback of shoot tips, and the formation of stem cankers. The specific manifestation of the disease is determined by the interaction between the *P. syringae* strain, the susceptibility of the host plant, and environmental factors (Córdova et al., 2023). At early stages, however, the disease may mimic the symptoms of fire blight (Billing, 2011), which complicates visual diagnosis and increases the risk of choosing inadequate control measures. Effective management of bacterial diseases in apple orchards depends on accurate diagnosis, as the recommended measures differ in timing and approach. Control of fire blight largely relies on timely sanitation measures/pruning to remove infected tissues (“Erwinia amylovora (ERWIAM)[Datasheet]— EPPO Global Database”; EPPO, 2022) and on interventions during the flowering period based on risk forecasting (*e.g.*, the Maryblyt/Cougarblight models) (Biggs & Turechek, 2010; DuPont, 2026). Therefore, incorrect identification of the causal agent may result in improper timing, ineffective treatments, and excessive use of plant protection products.

The initial aim of the study was an identification of bacterial isolates from apple trees from an orchard near Almaty using a combination of morphological, microbiological, and molecular-genomic methods, including whole-genome sequencing (WGS), to achieve accurate taxonomic identification and clarify species affiliation. Since classical microbiological traits and 16S rRNA do not always provide species-level differentiation of closely related Pseudomonas, taxonomic verification of the isolates was performed using WGS.

Despite the similarity of the symptoms observed, two different pathogens, *E. amylovora* and *P. syringae,* have been isolated and identified. A comprehensive diagnostic approach is warranted to achieve accurate taxonomic identification when classical symptom- and morphology-based methods have limited discriminatory power. This applies to both *P. syringae* and *E. amylovora*, for which WGS-based approaches are already being employed in comparative genomic studies (Holtappels et al., 2024; Maguvu et al., 2024). Whereas the genome of a *E. amylovora* isolate from Kazakhstan have been sequenced and assembled previously (Sadanov et al., 2024), the further expansion of the available data is important to elucidate the genetic variability of the bacterium on both individual and population levels. The isolates of *P. syringae* have not been sequenced previously in Kazakhstan. Thus, our results provide novel data on the genomes of the bacterial pathogens in the country and highlight the importance of combining morphological and molecular methods in the diagnosis of bacterial diseases of apple trees. This not only allows differentiation of pathogens with similar symptoms but also contributes to the development of targeted protection strategies aimed at reducing phytosanitary risks and preserving yield.

## Materials & Methods

### Collection of bacterial pathogen samples and isolation of pure cultures

Collection of apple tree samples was carried out in September, 2024 in a private orchard near Almaty city, Almaty region (N43°30′38″; E77°42′10″). Samples were collected using a targeted (symptom-based) sampling approach, prioritizing leaves with characteristic symptoms of bacterial diseases (necrotic spots on leaves and shoots). A total of 54 samples were collected and processed immediately upon the delivery to the laboratory facility.

For pathogen isolation, tissue fragments from the boundary between diseased and healthy areas were washed in a mild detergent solution (1% v/v, 1 min) and then rinsed three times with sterile distilled water. The resulting samples were homogenized (200 mg of tissue in 1 mL of Phosphate Buffered Saline (PBS) buffer), after which 30 µL of the suspension was dispersed over Petri dishes containing NSA medium (yeast extract 2 g/L, bactopeptone 5 g/L, NaCl 5 g/L, sucrose 50 g/L, agar 20 g/L; pH 7.0–7.2) using the sterile spatula. Five Petri dishes per sample were inoculated; additionally, one symptomless apple sample was used for inoculation, and one Petri dish was inoculated with 30 µL of pure PBS buffer as a negative control, before, after and during the inoculation process. The Petri dishes were further incubated at 25 °C for 2–4 days. The individual colonies were selected for further study and subcultured onto two types of nutrient media, NSA and King’s B medium (proteose peptone No. 3 20 g/L, glycerol 1% vol., K_2_HPO_4_ 1.5 g/L, MgSO_4_⋅7H_2_O 1.5 g/L, agar 15 g/L; pH 7.0–7.2) (Sadanov et al., 2024), using standard quadrant streaking method (Sanders, 2012). The cultures were further maintained by re-inoculation of the colonies on fresh media.

### Preliminary sequencing-based identification of apple bacterial pathogens

For the molecular identification of bacterial pathogens, isolated colonies (10 replicates) were used for DNA extraction with the FastPure Bacteria DNA Isolation Mini Kit-BOX 2 (Nanjing Vazyme Biotech, Nanjing, China) following the manufacturer’s protocol. First, an exploratory low-coverage genome sequencing was attempted using the MinION MK1b device (Oxford Nanopore Technologies, Oxford, United Kingdom) with the SQK-NBD114-96 kit. The libraries were prepared directly from the extracted genomic DNA following the manufacturer’s protocol. Second, amplification of 16S fragments (positions 340-806 of 16S rRNA) was performed using polymerase chain reaction (PCR) with universal primers for prokaryotic 16S amplification, Uni340F and Uni806R (Takai & Horikoshi, 2000). The reaction mixture (25 µL) contained 1 × Taq buffer, 0.2 mM dNTPs, 0.2 mM of each primer, 1 U of Taq DNA polymerase (Thermo Fisher Scientific, Waltham, MA, USA), and 2 µL of DNA template. PCR conditions: initial denaturation at 94 °C for 3 min; 30 cycles of 94 °C −30 s, 55 °C −30 s, 72 °C −1 min; final elongation at 72 °C for 10 min. Forward primer sequence: 5′-CCTACGGGRBGCASCAG-3′ (Uni340F); reverse primer sequence: 5′-GGACTACNNGGGTATCTAAT-3′ (Uni806R). Amplicons of the expected length 466 bp were tested in 1.5% agarose gel, and DNA concentration was measured using Qubit Flex (Invitrogen, Waltham, MA, USA) and the used for sequencing (MinION MK1b, SQK-NBD114-96, following the manufacturer’s protocol). Basecalling and barcode demultiplexing were carried out with Dorado v7.4.12.: the High-accuracy model Q9 minimal quality read quality threshold, barcode quality threshold 60; for the low coverage genome sequencing the reads of lengths less than 500 bp were discarded; for 16S rRNA sequencing only the reads of lengths between 200 and 500 bp were retained. The preliminary species assignment of the reads from the low-coverage genome sequencing has been done using Kraken 1.1.1 (Wood & Salzberg, 2014). Based on the results of the read identification of the sampled isolates by Kraken, the genomic sequences of *E. amylovora* (NR_041970.1) and *P. syringae* (NR_043716.1) were selected as the references for the mapping of 16S rRNA sequence reads. The mapping was performed using the minimap2 algorithm (Li, 2016) in Geneious Prime 2025.2.1 software. The obtained consensus sequences have been tested using the NCBI BLAST tool to identify the isolated species.

### Pathogenicity testing of bacterial isolates

Pure cultures of bacterial isolates were used to evaluate their pathogenicity on the leaves of various fruit crops. The experiment was conducted in five biological replicates using the following cultivars: plum “Stanley”, pear “Lesnaya Krasavitsa”, cherry “Lyubskaya”, and apple “Aport Alexandr”, “Burkhardt’s Renet”, and “Red Delicious”. Distilled water was used as a negative control. Prior to inoculation, the leaves were washed in a soap solution and thoroughly rinsed with distilled water. Leaf infiltration was carried out using a bacterial suspension prepared in agar-free NSA medium with an optical density of OD_6__0__0_ = 0.2–0.4, applied with a needleless syringe. After inoculation, the leaves were placed in a climate-controlled chamber: temperature 24–25 °C, relative humidity 80%. Incubation lasted for four days, with daily monitoring of the development and spread of infection symptoms.

After incubation, DNA was extracted from infected leaves, and the isolates were subjected to sequencing of the 16S rRNA region for molecular identification, as described above.

Additionally, pathogenicity was tested on pear fruits of the cultivar “Lesnaya Krasavitsa”, which served as a highly susceptible host (Kapytina et al., 2023). The fruits were washed in a soap solution, rinsed with distilled water, and surface-sterilized with 70% ethanol. Inoculation was performed by gently puncturing the fruit epidermis with a sterile pipette tip, followed by the introduction of the bacterial suspension. The inoculated fruits were incubated at 24–25 °C and 80% relative humidity. Daily observations were conducted to record disease progression and the appearance of bacterial exudate at the inoculation sites.

### Whole genome sequencing of the identified isolates

The suspected isolated bacterial cultures have been used for WGS using the FastaSeq300 system (GeneMind, Biosciences, Shenzhen, China). Raw paired-end sequencing reads were processed for downstream analysis using SOAPnuke (Chen et al., 2018) to remove adapter sequences, low-quality bases, and short reads (parameters “-n 0.001 -l 10 -q 0.5 –adaMR 0.25 –polyX 50 –minReadLen 200”). Read alignment, or mapping, was performed using BWA-MEM v.0.7.17 (Li, 2013) with default parameters. The reads were aligned against the reference genomes of *E. amylovora* and *P. syringae*: strain EaSmR (ASM4322886v1, GCF_043228865.1) (He et al., 2024) and strain Susan2139 (ASM1839437v1, GCF_018394375.1) (Lipps, Samac & Ishii, 2022), correspondingly. The resulting alignments were processed using SAMtools v.1.19.2 (Li et al., 2009) to generate sorted and indexed BAM files, followed by duplicate filtering. The mapping and coverage statistics have been collected using Stats and Coverage tools, and the assembled genome sequences have been obtained using Consensus tools of the SAMtools package. The identity of the consensus genome sequences was checked using the TYGS tool; the formula *d*_4_ for the digital DNA-DNA hybridization (dDDH) was selected as the best for interpretation, following the recommendations by the authors of the algorithm (Meier-Kolthoff & Göker, 2019). The average nucleotide identity (ANI) percentages between the obtained sequences and the reference genomes of *Erwinia* and *Pseudomonas* have been calculated using FastANI software (Jain et al., 2018). The ANI value 95% was selected as the threshold for the species definition, as recommended by Richter & Rosselló-Móra (2009).

The whole genome phylogenetic analysis was performed using the universal bacterial core genes (UBCG) (Na et al., 2018) pipeline with MAFFT (Katoh et al., 2002) for multiple sequence alignment and RaxML (Stamatakis, 2014) for phylogenetic tree inference (GTR+CAT model, default for the pipeline, according to the built-in user manual). For *Pseudomonas,* the available genomic sequences of *P. syringae* as well as the reference genomes of other species of the genus were used for comparison; for *Erwinia,* all available *E. amylovora* genomes were used with the reference genome of *E. pyrifolia* as an outgroup (Table S2). The neighbor joining tree with 1,000 replicates was calculated using the concatenated sequences of 92 UBCG genes using MEGA11 with the default parameters (Tamura-Nei model, uniform rates among sites) (Tamura, Stecher & Kumar, 2021). FigTree (Rambaut, 2018) was used for the visualization of phylogenetic trees. For the analysis, a set of complete bacterial genomic sequences of the bacterial strains and isolates within the identified species and genera has been retrieved from the NCBI Genomes database.

For the analysis of CRISPR repeat regions in the *E. amylovora* isolates, the reads corresponding to these regions were extracted from the raw sequencing reads and assembled using MetaCRISPR (Lei & Sun, 2016). Additionally, the CRISPR regions were extracted from the genome sequence of the local isolate E22 by Sadanov et al. (2024), for comparison, using CRISRPCasFinder (Couvin et al., 2018). The obtained sequences were identified as CRISPR1, CRISPR2, and CRISPR3 repeat regions using BLAST search, compared to all corresponding sequences available in NCBI GeneBank database by manual alignment to ensure correctness of the assembly, and analysed based on the known data on CRISPR spacers in *E. amylovora* (Rezzonico, Smits & Duffy, 2011).

### Data summary

All obtained data have been published in the NCBI databases. The genomic data obtained in the study as well as the raw sequencing reads are available under the BioProject accession PRJNA1314673. The 16S rRNA sequences are available in the NCBI GenBank database under the accession numbers PX502605–PX502609. The sequences of CRISPR repeat regions are available in the NCBI GenBank database under the accession numbers PX842731–PX842736.

## Results

### Preliminary identification of pathogens by morphological and molecular methods

**Figure 1:**
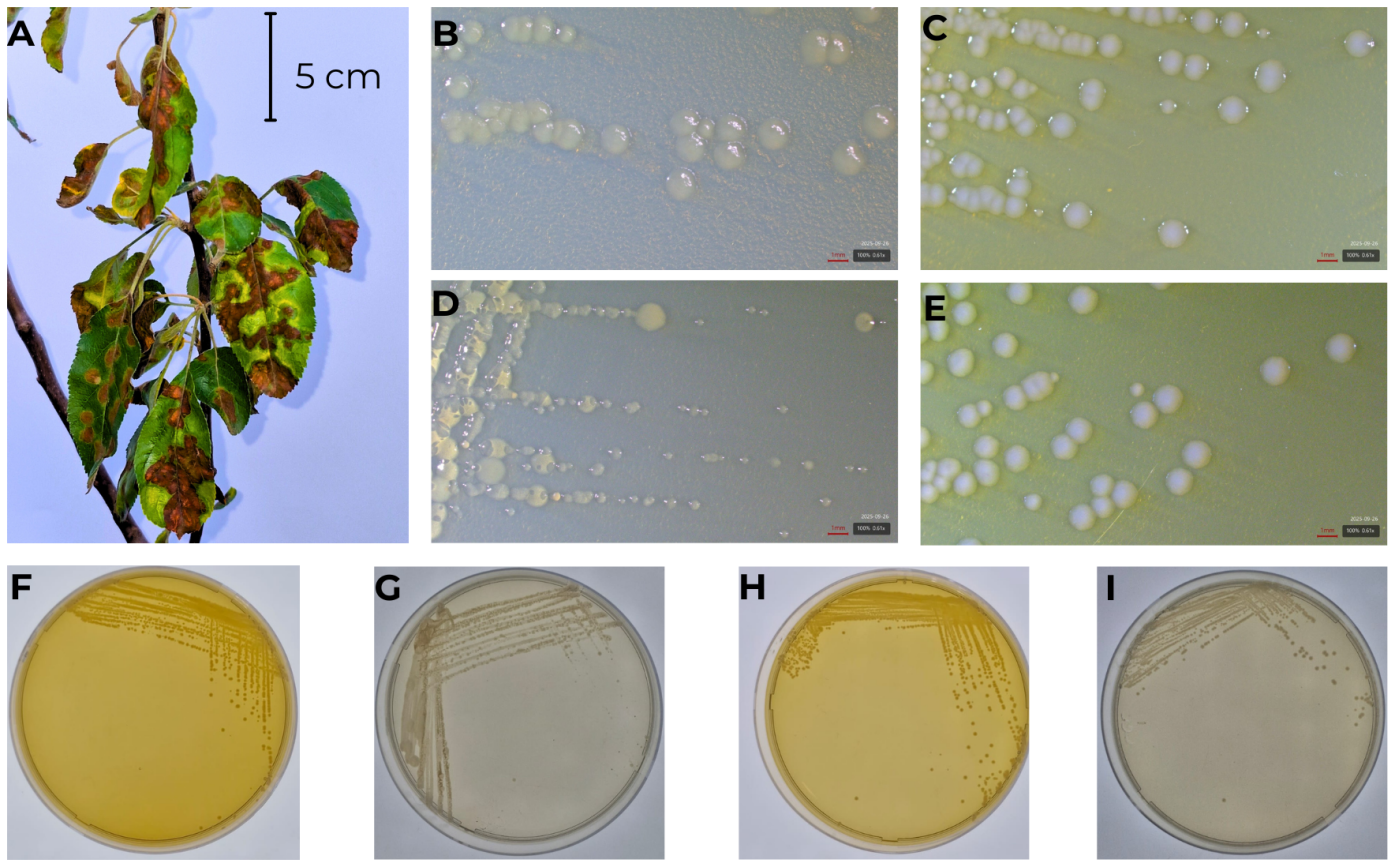
Symptoms and culture of bacterial pathogens of apple trees. (A) A sample of apple leaves with the symptoms of bacterial infection; (C, F) *Erwinia amylovora* on the King’s medium; (B, G) *Erwinia amylovora* on the NSA medium; (E, H) *Pseudomonas syringae* on the King’s medium; (D, I) *Pseudomonas syringae* on the NSA medium.

From the 54 apple tree samples exhibiting leaf necrosis, five bacterial isolates have been successfully obtained and cultured on King’s B and NSA media. On both selective media, the presumable pathogens displayed similar morphological patterns: consistent growth characterized by the formation of colonies that merged with each other. On King’s B medium, the studied *Pseudomonas* strains did not exhibit fluorescence. The colonies were whitish, round, dome-shaped, smooth, and mucilaginous (Fig. 1). The three of five cultures on both culture media displayed the presence of the viscous slime-like substance in the colonies corresponding. All five isolates have been confirmed having negative Gram-staining status by conventional microscopic examination.

The low coverage genome sequencing and targeted sequencing of the 16S rRNA gene region using the Oxford Nanopore MinION platform were attempted for the preliminary species identification. The initial classification of sequencing reads by the Kraken algorithm identified two isolates as *Erwinia amylovor* a and three as *Pseudomonas syringae* spp. group (*Pseudomonas syringae sensu lato*), and thus these isolates designated as Ea08KZ and Ea09KZ and Ps01KZ, Ps02KZ, and Ps03KZ, correspondingly (Table 1). Further, the consensus 16S rRNA sequences were obtained by the read mapping against the reference sequences (NR_041970.1 for *E. amylovora* and NR_043716.1 for *P. syringae*) and analyzed using the BLAST search. Due to the uneven mapping of the amplicon termini, the aligned 16S sequences were trimmed to 452 bp, for consistency. Whereas *E. amylovora* sequences resulted in unambiguous species hits, the *P. syringae* s.l. isolates resulted in less conclusive results, with the mixed hits for *P. syringae,* related *Pseudomonas* species, and the *Pseudomonas* isolates of the unclassified species (Table 1). A significant share of BLAST hits belonged to the unclassified *Pseudomonas* spp. isolates in all three isolated. The isolate Ps02KZ demonstrated highest number of hits for *P. syringae* hits, and isolate Ps03KZ had 11 hits for this species, next to 46 hits for unidentified *Pseudomonas* spp. Considering this and the previous knowledge about distribution of *P. syringae* in apple trees as the main agent of *Pseudomonas* infection, we assumed this species as the primary candidate to be further validated by the whole genome sequencing and phylogenetic analysis. However, the observed uncertainty could lead to low sequencing quality, which necessitated the use of methods with broader genomic coverage.

**Table 1:** Summary of the bacterial species identification based on the low coverage genome sequencing and targeted 16S rRNA gene sequencing.

**Isolate**	**Species and genera with highest read percentage identified by Kraken based on low coverage genome sequencing reads**	**Best numbers of BLAST hits by species for 16S rRNA**			
		**Species**	**Number of hits**	**Average identity, %**	**Average hit score**
Ea08KZ	*Erwinia amylovora* 49.45% *Erwinia* 10.85%	*Erwinia amylovora*	103	98.67	811
		*Erwinia papayae*	1	98.67	811
Ea09KZ	*Erwinia amylovora* 51.19% *Erwinia 14.13%*	*Erwinia amylovora*	101	99.12	819
Ps01KZ	*Pseudomonas syringae* group 49.98% *Pseudomonas* 40.37%	Unclassified *Pseudomonas*	22	99.78	832
		*Pseudomonas azotoformans*	33	99.78	832
		*Pseudomonas fluorescens*	4	99.78	832
Ps02KZ	*Pseudomonas syringae* group 45.20% *Pseudomonas* 40.62%	*Pseudomonas syringae*	26	99.55	832
		Unclassified *Pseudomonas*	19	99.78	832
		*Pseudomonas congelans*	4	99.78	832
Ps03KZ	*Pseudomonas syringae* group 30.20% *Pseudomonas* 34.77%	*Pseudomonas syringae* group	46	99.78	830
		*Pseudomonas syringae*	11	99.78	830
		*Pseudomonas coronafaciens*	13	99.78	828

### Pathogenicity testing of *Erwinia amylovora* and *Pseudomonas* spp. isolates

**Figure 2:**
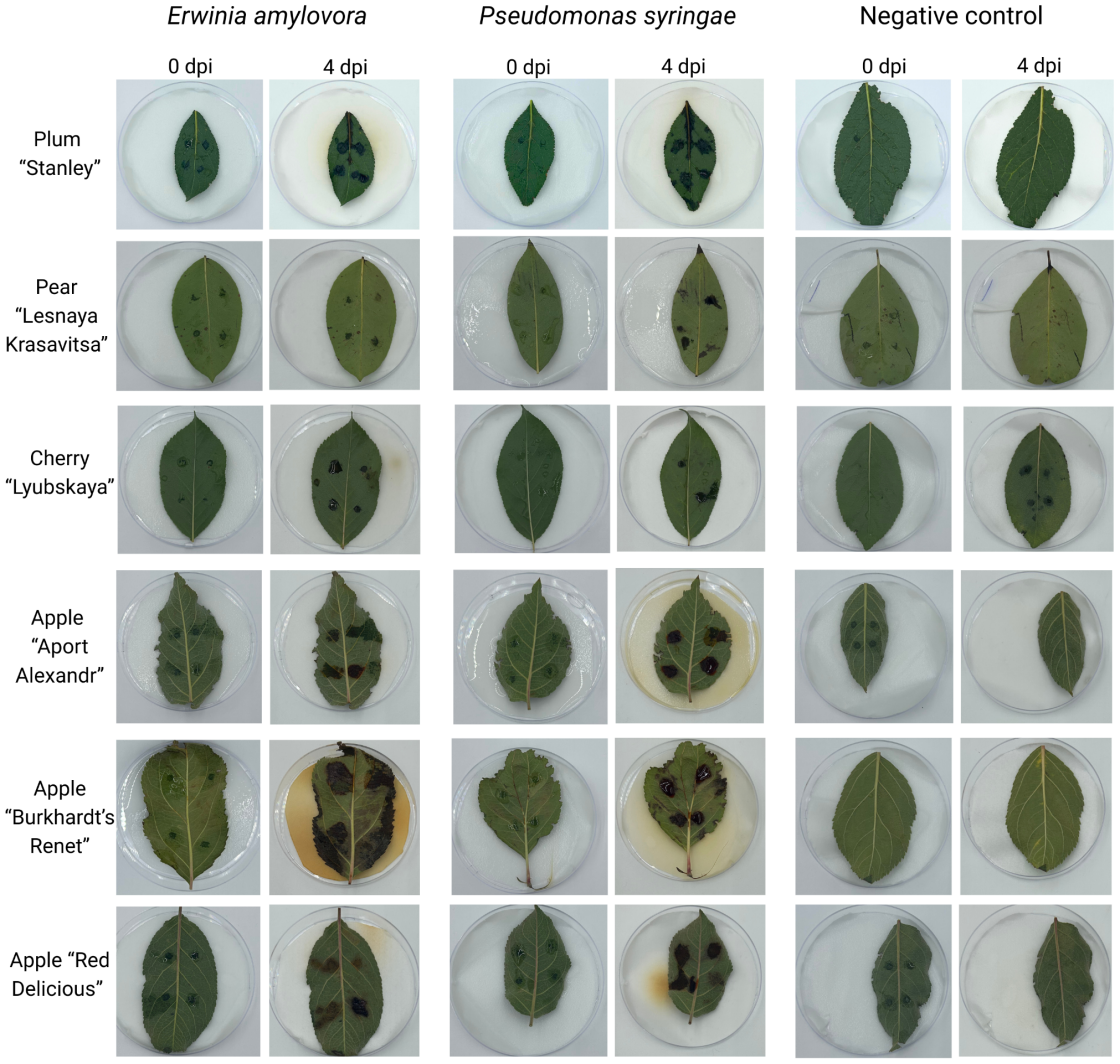
Results of inoculation of the isolated leaves of fruit tree crops by the obtained bacterial isolates. Dpi, days post inoculation. See also Table S2 for complete images for all isolates during the observation period.

The pathogenicity test on leaves results demonstrated varying degrees of susceptibility of fruit crops to the studied isolates of *Erwinia amylovora* and *Pseudomonas* spp (Fig. 2; Table S1). The most susceptible was the apple cultivar “Burkhardt’s Renet”, in which the first disease symptoms appeared on the leaves 24 h after inoculation. Thirty-six hours after infection, a characteristic secretion of mucous exudate was observed on the leaf surface treated with *Pseudomonas* spp. isolates.

In the plum cultivar “Stanley”, the development of symptoms occurred significantly more slowly. The first signs of infection appeared 72 h after inoculation and were manifested by tissue browning and the formation of localized necrotic lesions. Subsequently, the appearance of a mucilaginous exudate at the inoculation sites indicated the progression of the bacterial process. On the leaves of the cherry cultivar “Lyubskaya” and the pear cultivar “Lesnaya Krasavitsa”, no symptoms were observed at all.

**Figure 3:**
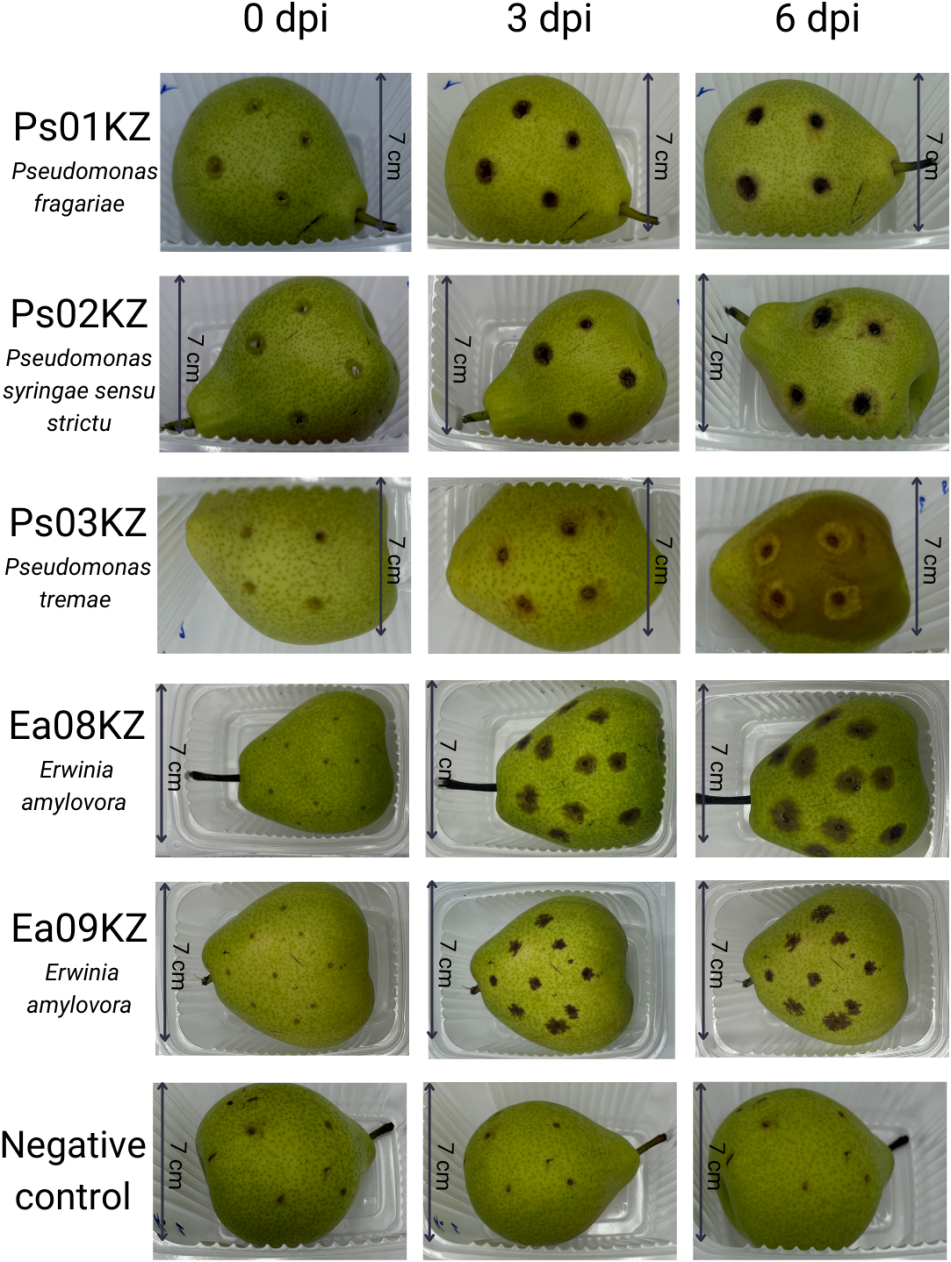
Results of pear fruit inoculation by isolates of *Erwinia amylovora* and *Pseudomonas syringae s.l*. Dpi, days post inoculation.

In tests performed on fruits of the pear cultivar “Lesnaya Krasavitsa”, symptoms typical of *E. amylovora* began to appear on the third day after inoculation in the form of watery, slightly sunken spots, which quickly turned brown and then blackened (Fig. 3). Symptoms observed upon infection with *Pseudomonas* spp. isolates differed in nature and dynamics. Dark brown, later blackened necrotic spots with a watery halo along the periphery developed on the fruit surface. In some cases, slight tissue depression and loss of turgor were observed.

After the documentation of the infection symptoms, the leaves of “Burkhardt’s Renet” were used to isolate DNA and perform PCR and sequencing of 16S rRNA partial gene, and the sequencing results have been compared with the initially obtained 16S sequences. As a result, the replicated sequences for isolates Ea08KZ and Ea09KZ displayed sequence identities 99.78% (one mistake per 452 bases) and 100% compared to the initial sequences. For three *Pseudomonas* isolates, the identity varied from 99.56% (two mistakes per 452 bases) for isolate Ps02KZ to 99.12% (four mistakes per 452 bases) for isolates Ps01KZ and Ps03KZ. Thus, even considering the certain mistake rate produced by the Nanopore sequencing, the results of re-sequencing confirm the identity of the inoculated isolates in accordance with R. Koch’s postulates. The obtained results indicate high pathogenic activity of the studied isolates and confirm cultivar-specific differences in the susceptibility of fruit crops to bacterial pathogens.

### Whole-genome sequencing and analysis of *Erwinia amylovora* isolates

The whole genome sequencing of two *E. amylovora* isolates (Ea08KZ, Ea09KZ) and three *Pseudomonas* isolates (Ps01KZ, Ps02KZ, Ps03KZ) has produced, after the quality filtering, about 9.5–13.8 million paired end reads of length 200 and average quality 39.2 per isolate (Table 2). The reference genomes for the read mapping have been selected based on the preliminary identification based on 16S sequences. Reads from *E. amylovora* were aligned to the reference genome assembly ASM4322886v1 (NCBI Genome accession GCA_043228865). Reads from *P. syringae* were aligned to the reference genome assembly *P. syringae* Susan2139 strain ASM1839437v1 (NCBI Genome accession GCA_018394375). *Erwinia amylovora* produced high quality alignments with coverage above 99.5% in the chromosome and 99.9–100% in plasmid pEA29, the mean coverage depth above 100, and the average mapping quality about 59 for the chromosome and 60 for plasmid. The ANI values for both isolates was near 100% (99.97%) allowing their unambiguous assignment to *E. amylovora*. Type Strain Genome Server (TYGS) analysis also identified two isolates as *E. amylovora* with a high confidence: dDDH scores 99.9% with Confidence Interval (CI) [99.8–99.9]% (Table 3).

**Table 2:** Summary of the whole genome read mapping of bacterial isolates against the reference sequences.

**Isolate**	**Total read number/average quality (PHRED)**	**Reference species**	**Reference assembly accession**	**Genome segment**	**Mapped reads**	**Mapping coverage, percent**	**Average coverage depth**	**Average mapping quality**
Ea08KZ	9,461,292/39.2	*Erwinia amylovora*	GCA_043228865	Chromosome	8,830,929	99.8	339.2	59
				Plasmid pEA29	27,391	100	134.8	60
Ea09KZ	11,729,034/39.2	*Erwinia amylovora*	GCA_043228865	Chromosome	11,052,898	99.6	431.3	58.9
				Plasmid pEA29	35,957	99.9	179.1	60
Ps01KZ	12,358,254/39.3	*Pseudomonas syringae*	GCA_018394375.1	Chromosome	9,929,715	86.7981	236.595	58.7
Ps02KZ	13,796,400/39.2	*Pseudomonas syringae*	GCA_018394375.1	Chromosome	11,605,402	90.1	282.6	59.1
Ps03KZ	13,542,476/39.2	*Pseudomonas syringae*	GCA_018394375.1	Chromosome	8,093,441	73.9	165.8	57
		*Pseudomonas tremae* ^*^	GCA_023278125^*^	Chromosome	12,590,801	91	318.3	59.5

**Notes.**

* Realignment based on the species re-assignment based on ANI results (Table 3).

**Table 3:** Comparison of the read mapping statistics using alternative reference genomes for isolates Ps01KZ and Ps03KZ.

**Isolate**	**Best matching species/strains**	**ANI, %**	**dDDH d4, %**
Ea08KZ	*Erwinia amylovora* CFPB 1232 (type)	99.9594	99.9
	*Erwinia pyrifoliae* DSM 12163 (type)	90.7434	41.0
	*Erwinia piriflorinigrans* CFBP 5888 (type)	85.6202	30.0
Ea09KZ	*Erwinia amylovora* CFPB 1232 (type)	99.9601	99.9
	*Erwinia pyrifoliae* DSM 12163 (type)	90.7536	41.0
	*Erwinia piriflorinigrans* CFBP 5888 (type)	85.6203	30.0
Ps01KZ	*Pseudomonas fragariae* 17 (type)	98.6172	88.7
	*Pseudomonas syringae* KCTC 12500 (type)	95.144	62.6
	*Pseudomonas cerasi* 58 (type)	94.5503	59.1
Ps02KZ	*Pseudomonas syringae* KCTC 12500 (type)	98.1272	85.0
	*Pseudomonasfragariae* 17 (type)	95.119	61.9
	*Pseudomonas cerasi* 58 (type)	94.5835	59.5
Ps03KZ	*Pseudomonas tremae* PA-1-10f (RefSeq)	97.4693	No data
	*Pseudomonas syringae* KCTC 12500 (type)	88.3506	34.5
	*Pseudomonas avellanae* BPIC 631 (type)	86.6761	34.1

**Figure 4:**
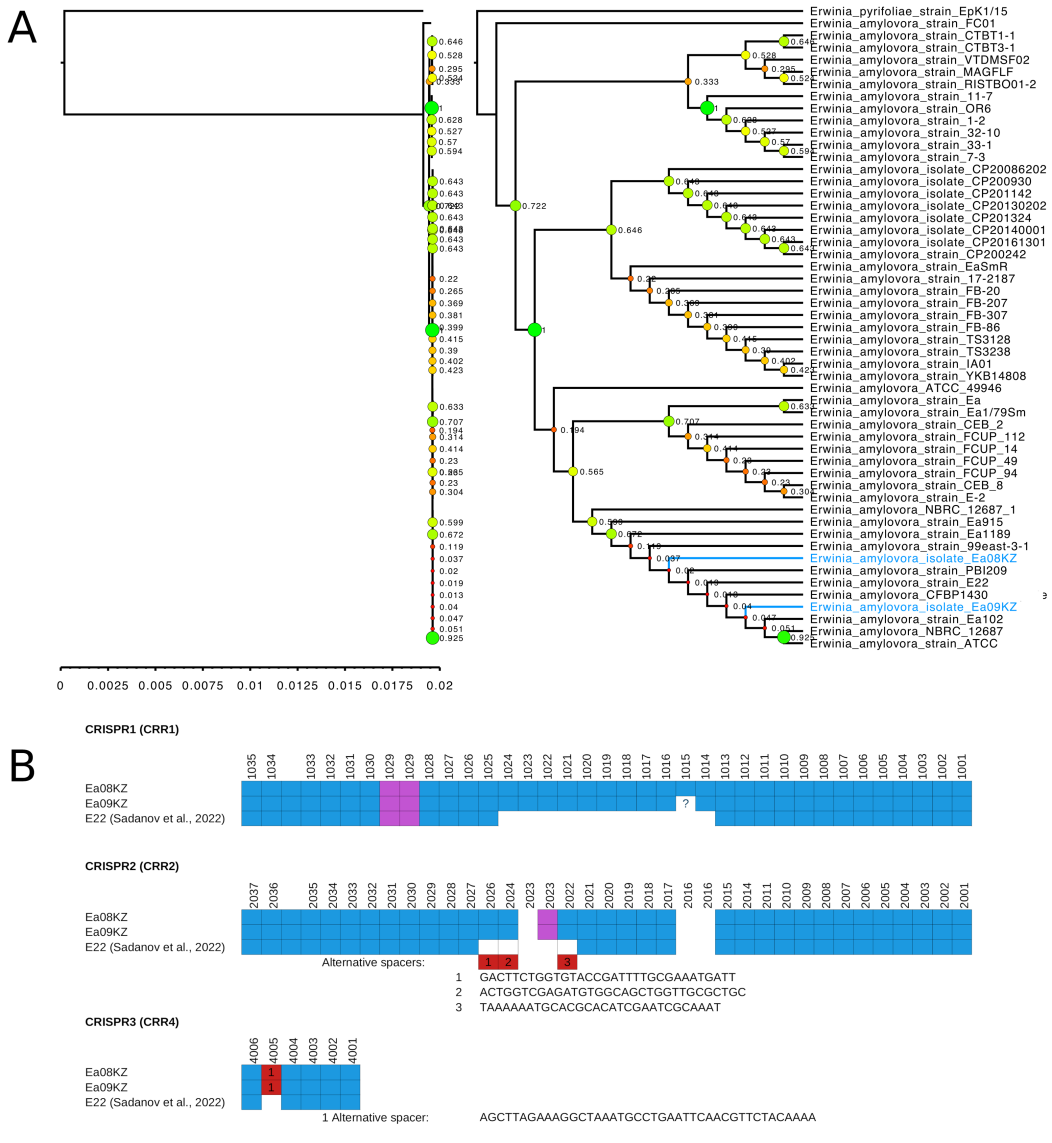
Phylogenetic analysis of *Erwinia amylova* isolates Ea08KZ and Ea09KZ in comparison to the available genomes. Neighbor joining tree of the concatenated sequences of 92 core genes; numbers and circles at the nodes indicate the bootstrap support (left); consensus tree of *Erwinia* genomes built from 92 individual maximum likelihood trees for the UBCG gene set; numbers and circles at the nodes indicate the node support rate by individual gene trees (right).

For the phylogenetic analyses, the available genomic sequences of *E. amyovora* and *P. syringae*, as well as other species of the corresponding genera, have been retrieved (Table S2). Phylogenetic analysis based on whole-genome sequences showed that the isolates from our study (Ea08KZ and Ea09KZ, highlighted in blue) along with the local isolate E22 (Sadanov et al., 2024) formed a cluster with a selection of isolates/strains with different geographic origin: CFPB1430 (France), Ea1189 (USA), Ea915, 99east-3-1, PBI209 (Xinjiang, China), NBRC12687[=CFPB1232], ATCC 15580 (UK) (Fig. 4A). Whereas this cluster displayed the bootstrap value of 0.599, its internal topology had low support making more precise resolution impossible. Notably, the newly identified isolates were clearly different from the isolate E22 from the same Almaty region, indicating the presence of variable strains of *E. amylovora* in the region.

Due to the limited variability of *E. amylovora* isolates based on 92 UBCG genes, we have additionally analysed CRISPR regions of our isolates (Fig. 4A). The sequences were extracted and assembled from the raw reads and identified by BLAST as the CRISPR1 (CRR1), CRISPR2 (CRR2), and CRISPR3 (CRR4, according to Rezzonico, Smits & Duffy, 2011) regions of *E. amylovora*. Also, the CRISPR regions were extracted from the available genome of isolate E22 for comparison. The distribution of known CRISPR spacers was interpreted according to the published data (Rezzonico, Smits & Duffy, 2011; Kurz et al., 2021; Rezzonico et al., 2024). Based on the duplication of the spacer 1029 (GAT TGC GCA TGA GCA CTG AAA TTG TTC ACA GC), both isolates Ea08KZ and Ea09KZ were identified as belonging to the A haplotype. Isolate Ea09KZ missed spacer 1015 (ACG ATT TGC CTG AAA CCT CAA CGA AGT TCG AC); however, since the absence of this sole spacer in the A group haplotypes has not been previously reported, we cannot be sure that this was not an artefact of sequencing. Unlike Ea08KZ and Ea09KZ, the isolate E22 corresponded to the subtype Ã according to Rezzonico et al. (2024), which comprised the A haplotype with the absent region on spacers 1014-1024.

For CRIPSR2, based on the spacer composition, isolates Ea08KZ and Ea09KZ were identified as belonging to haplotype t with absent spacer 2016 (CTG GAG CAT GAG ACG AAA TCG GGG GTA GTG CT) and the single copy of spacer 2023 (GCT ACT ACG TGT ACG CAC AGC CGC TGG CCA GT). Isolate E22 demonstrated the absence of both spacers 2016 and 2023; it also contained three unique spacers at positions corresponding to spacers 2022, 2024, and 2026 in isolates Ea08KZ and Ea09KZ. Based on the classification by Rezzonico et al. (2024), we consider this isolate a modification of haplotype t with the replacement of the region 2022-2026 with three novel spacers; here, we will further refer to it as t*.

Isolates Ea08KZ and Ea09KZ have shown the presence of a novel spacer (AGC TTA GAA AGG CTA AAT GCC TGA ATT CAA CGT TCT ACA AAA) in place of spacer 4005 previously found in isolates from *Rubus* species (Rezzonico, Smits & Duffy, 2011); the same spacer was also found in isolate RBA4 from *Rubus* (Michigan, USA; NCBI accession number JQ796601.1) with 100% identity with isolates Ea08KZ and Ea09KZ. Isolate E22 had the typical composition for *E. amyovora* (*α* haplotype).

Based on the CRISPR analysis, the isolates Ea08KZ and Ea09KZ were classified as belonging to At group with rare CRR4 variant. The previously sequenced isolate E22 demonstrated novel haplotype Ãt* with the CRR4 haplotype typical for *E. amylovora*.

### Whole-genome sequencing and analysis of *Pseudomonas* spp. isolates

The read alignments against the *P. syringae* reference genome demonstrated higher variation in the mapping statistics. The initial identity analysis based in ANI and dDDH allowed to identify isolate Ps01KZ as *P. fragariae,* isolate Ps02KZ as *P. syringae sensu strictu,* and isolate Ps03KZ as *P. tremae* (Table 3). For isolates Ps01KZ and Ps02KZ two species, *P. syringae* and *P. fragariae*, had ANI satisfying identification threshold 95% (Richter & Rosselló-Móra, 2009) thus the species with the higher ANI and dDDH values were assigned. For *P. tremae,* the identification was based on ANI = 97.47%; the dDDH was not obtained as *P. tremae* was not present in the TYSG database. Based on this identification, we have performed re-alignment of the reads from isolate Ps03KZ against the reference genome of *P. tremae* (GCA_023278125) (Hulin et al., 2023) to clarify the species identity. As a result, the mapping statistics indeed have shown improvement of the whole assembly (Table 2): the coverage has increased from 73.9% to 91%, the mean coverage depth and quality also have been improved. For isolate Ps01KZ (*P. fragariae*) the only available assemblies by Marin et al. (2024), are currently limited to the contig level and thus the re-alignment of this isolate against them would not improve the obtained genome sequence.

**Figure 5:**
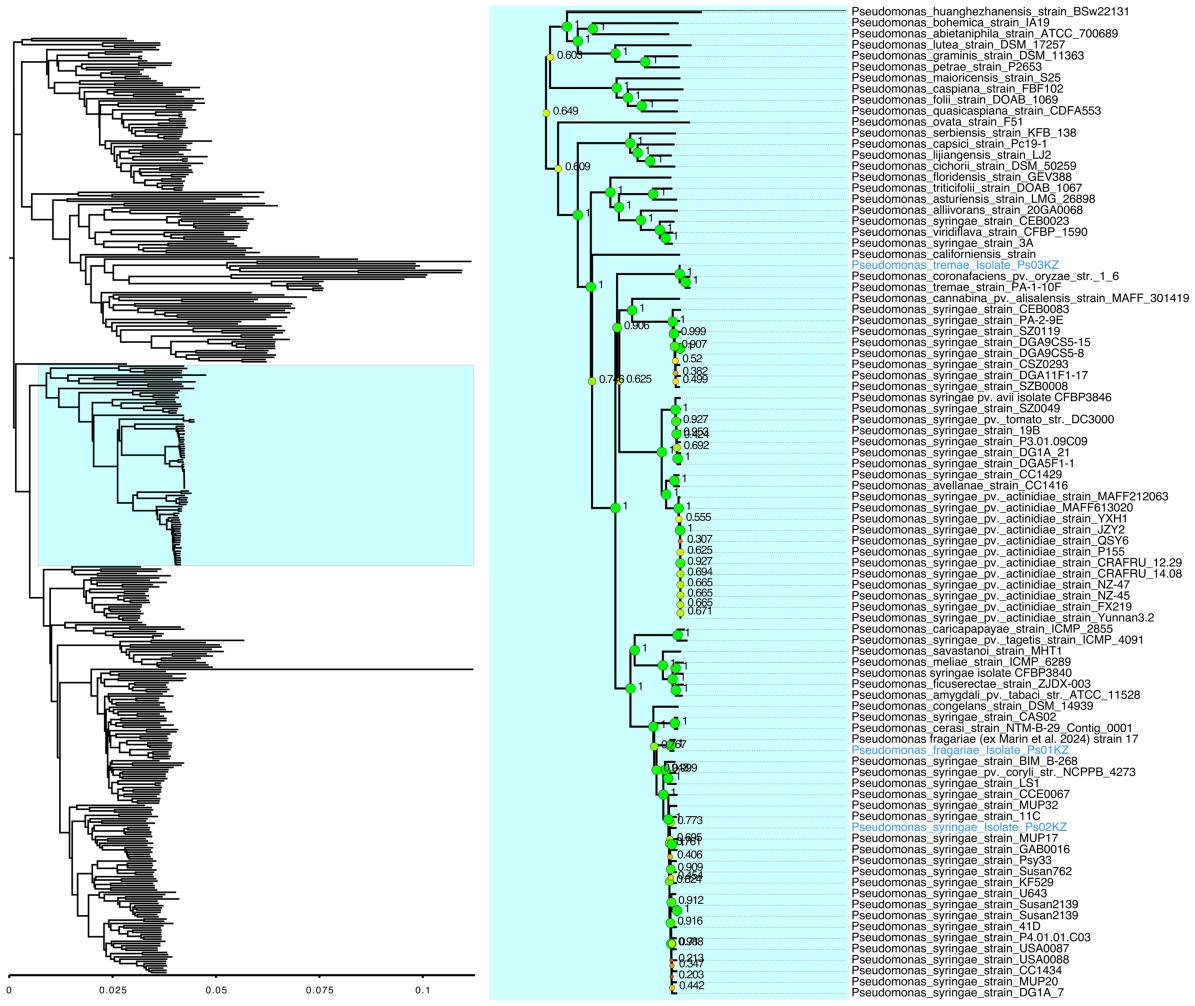
Phylogenetic analysis of *Pseudomonas syringae s.l.* isolates Ps01KZ, Pa02KZ, and Ps03KZ in comparison to the available *Pseudomonas* genome. (A) neighbor joining tree of the concatenated sequences of 92 core genes; numbers at the nodes indicate the bootstrap support; (B) The subtree of the consensus tree of *Pseudomonas* genomes built from 92 individual maximum likelihood trees for the UBCG gene set. Numbers at the nodes indicate the node support rate by individual gene trees. See also Figs. S1 and S2 for the complete tree images.

The patterns demonstrated by the phylogenetic analysis of *Pseudomonas* isolates were consistent with the species identification (Fig. 5, Fig. S1). The *P. syringae* genomes available in the NCBI Genome database formed a distinct cluster among the closely related species referred as *Pseudomonas syringae* species group (Baltrus, 2016). Isolate Ps01KZ was placed close to the *P. fragariae* strain 17 (type strain). Isolate Ps02KZ was placed within the cluster including *P. syringae* strains from Australia (MUP17, MUP32), France (11c, CCE0067, GAB0016, Psy33), USA (Susan762, Susan2139, USA0087), *etc.*

## Discussion

Our results show that symptom assessment and cultivation-based phenotyping (including colony morphology and fluorescence on King’s B medium) may be insufficient for reliable identification of closely related bacterial species. In addition, 16S rRNA gene sequencing often does not provide sufficient resolution for members of the genus *Pseudomonas*. Therefore, higher-resolution methods, including whole-genome sequencing, are required for accurate taxonomic identification. In the investigated infected apple samples, isolates identified as *E. amylovora* and *Pseudomonas* strains produced overlapping symptoms on the original host trees and in inoculation experiments and showed similar colony morphology on the culture media used, which can complicate reliable species-level differentiation based solely on symptomatology and morphology. Traditionally used targeted 16S rRNA sequencing has also shown limited applicability. As we have seen, whereas the determination of *E. amylovora* using 16S rRNA sequence was unambiguous, the classification of *Pseudomonas* spp. was not definitive. This is consistent with previous studies showing that the 16S rRNA gene often lacks sufficient resolution for species-level identification within closely related bacterial taxa, including *Pseudomonas* (Anzai et al., 2000; Mulet, Lalucat & García-Valdés, 2010; Thompson et al., 2015). These limitations necessitate the use of more precise methods based on whole genome sequencing. Species- and lineage-level identification within *Pseudomonas* is practically important because taxa may differ in host range, epidemiology, and pathogenicity, which can affect the timing and expected effectiveness of integrated management in orchards and nurseries (Kennelly et al., 2007). In particular, copper-based bactericides are widely used against *Pseudomonas* diseases (Moore & Pscheidt, 2023), yet copper resistance has been reported in *P. syringae* populations (Cazorla et al., 2002), which may reduce treatment efficacy and increase the risk of ineffective or excessive applications. Therefore, higher-resolution diagnostics (beyond 16S rRNA) supports more targeted recommendations and more reliable interpretation in an applied context.

The obtained genome sequencing results provide new data on the bacterial pathogens of apple trees in Almaty region, Kazakhstan. The observed CRISPR repeat patterns within *E. amylovora* isolates were consistent with the previous data on distribution of the At haplotype group in Almaty region (Maltseva et al., 2023; Maltseva et al., 2025; Rezzonico et al., 2024). In addition to the available *E. amylovora* isolate E22 genome from the region (Sadanov et al., 2024), the novel genomic sequences of two isolates have been obtained and expected to become a part of knowledge about the variability of the bacterium in the region. The high degree of genetic similarity between the *E. amylovora* isolates studied here and comparable genomes from other countries indicates their close relatedness and assignment to a widely prevalent clonal group. At the same time, published data indicate that *E. amylovora* overall is characterized by a combination of a large conserved core genome and a variable genomic component, and that the low variability observed in individual datasets often reflects population structure and the dominance of epidemic clones (Mann et al., 2013; Talhi et al., 2024; Donat et al., 2007). It has also been shown that even under overall genomic conservatism, the species comprises distinguishable lineages/clades and variable elements (plasmids and mobile genetic elements) that are relevant to epidemiology and resistance (Parcey et al., 2020; Mendes et al., 2024). At the regional level, outbreaks may be characterized by the predominance of particular genotypes/lineages with limited variability, as demonstrated by both WGS-based approaches and multiple locus variable-number tandem repeat (VNTR) analysis (MLVA) typing (Bühlmann et al., 2014; Zeng et al., 2018; Albanese et al., 2022).

The genomic sequences of *P. syringae sensu lato* have shown that even within the close population present in a single orchard the various even species could circulate at one host. For isolate, Ps03KZ, genomic sequencing suggested assignment as *P. tremae* which has not been previously reported in Kazakhstan; its primary host, *Trema orientalis* (Gardan et al., 1999), is a tropical plant (Barstow, 2017) and thus also does not appear in the moderate climate of the region. Its isolation from an apple tree was also surprising as no previous studies reported *P. tremae* infecting apple and other fruit tree crops. Another isolate, Ps01KZ, belonging to the recently described species *P. fragariae* closely related to *P. syringae* (Marin et al., 2024), have been identified and sequenced for the first time outside the USA. Thus, further studies of this isolate as well as other available and newly discovered isolates of *P. syringae s.l.* in the region may reveal new findings about the pathogen and its variability. In the present study, whole-genome data were used primarily for reliable taxonomic verification and for placing the isolates within the existing classification framework, since within the *P. syringae s.l.* complex, delimiting closely related taxa at the species/subspecies/pathovar level is often challenging (Gomila et al., 2015; Gomila et al., 2017; Baltrus, McCann & Guttman, 2017). The studies of the growing spectra of the available whole genome data become the crucial tool of the systematics of the genus, specifically closely related species of the *Pseudomo nas* genus (Tran, Savka & Gan, 2017; Saati-Santamaría et al., 2021).

## Conclusions

We present results of a screening of an apple orchard near Almaty and provide novel genomic data on two local isolates of *E. amylovora* and the genomic sequence of *P. syringae s.l*.. The genomic sequences of *P. syringae* have been obtained for the first time in Kazakhstan. For the first time, *P. tremae* and *P. fragariae* were identified in the country. Our results highlight the complicated classification of the species complex *P. syringae* and show the importance of further extended in-depth investigation of the *P. syringae sensu lato* isolates in the region to better understand their distribution and influence on wild and domestic apple and other fruit trees. In general, the obtained data not only are important for the understanding and control of bacterial pathogens of apple in Kazakhstan but also will provide a contribution to the global knowledge about genomic variability of the studied bacteria. Further studies involving functional genomic analysis and experiments on the host-pathogen interaction will deepen the understanding of the diversity of the local fruit tree pathogens in Kazakhstan and Central Asia and thus fill the gap of knowledge about bacterial isolates in the region.

##  Supplemental Information

10.7717/peerj.21078/supp-1 Supplemental Information 1 Partial 16S rRNA sequences of two *Erwinia amylovora* and three *Pseudomonas syringae s.l.* isolates

10.7717/peerj.21078/supp-2 Supplemental Information 2 Neighbor joining tree for *Pseudomonas* species built using concatenated sequences of 92 UBCG genes

10.7717/peerj.21078/supp-3 Supplemental Information 3 Maximum likelihood phylogenetic tree for *Pseudomonas* species built using consensus of 92 individual trees for the UBCG gene set

10.7717/peerj.21078/supp-4 Supplemental Information 4 Results of inoculation of the isolated leaves of the fruit crops by isolates of *Erwinia amylovora* and *Pseudomonas syringa s.l.*

10.7717/peerj.21078/supp-5 Supplemental Information 5 NCBI Genome accession of *Erwinia* and *Pseudomonas* species used for phylogenetic analysis

10.7717/peerj.21078/supp-6 Supplemental Information 6 Results of TYSG species assignment based on genomic sequences of the studied isolates

## Additional Information and Declarations

### Data Availability

The following information was supplied regarding data availability: The raw sequencing data obtained in the study are available at NCBI: PRJNA1314673. The 16S rRNA sequences are available at NCBI GenBank: PX502605–PX502609. The sequences of CRISPR repeat regions are available at NCBI GenBank: PX842731–PX842736.
