# Supplementary figures and images for: Screening and genomic identification of the bacterial pathogens from an apple orchard in Kazakhstan
Source: PeerJ. 2026 Jun 23;14:e21078. doi: 10.7717/peerj.21078 (PMC13308539; doi:10.7717/peerj.21078)

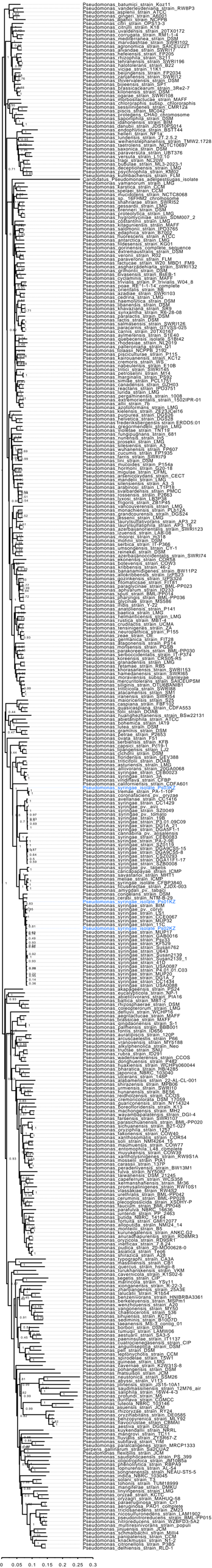

Supplement: Supplemental Information 2 [file peerj-14-21078-s002.pdf]

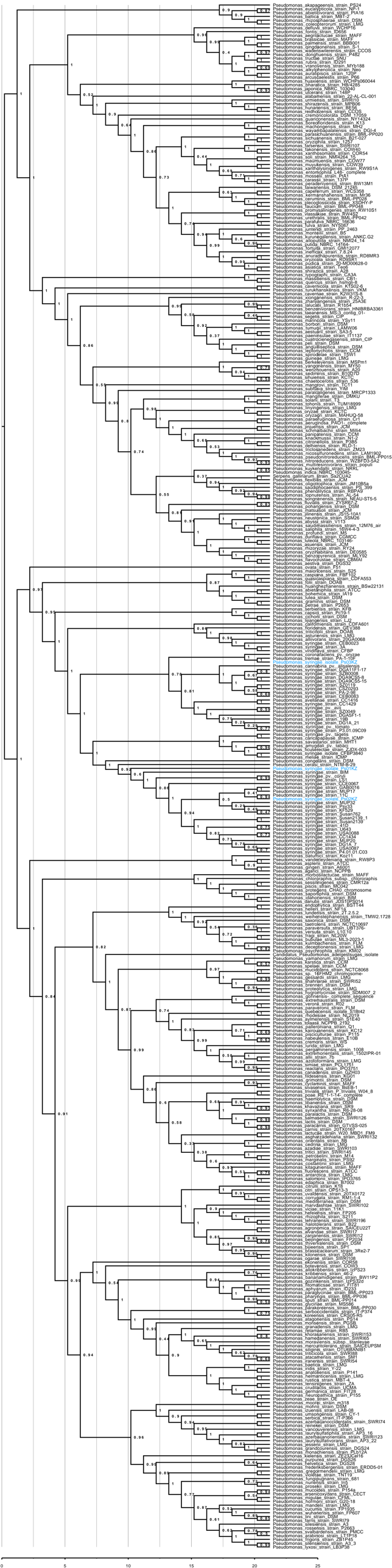

Supplement: Supplemental Information 3 [file peerj-14-21078-s003.pdf]
