# Supplementary material for: Screening and genomic identification of the bacterial pathogens from an apple orchard in Kazakhstan
Source: PeerJ. 2026 Jun 23;14:e21078. doi: 10.7717/peerj.21078 (PMC13308539; doi:10.7717/peerj.21078)
Supplement: Supplemental Information 4 [file peerj-14-21078-s004.pdf]

0 dpi		
Ps01KZ	plum “Stanley”	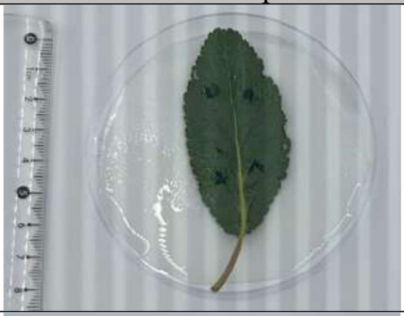
	pear “Lesnaya Krasavitsa”	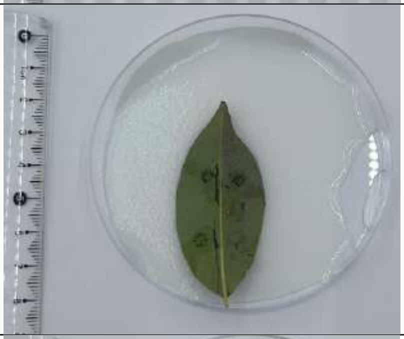
	cherry “Lyubskaya”	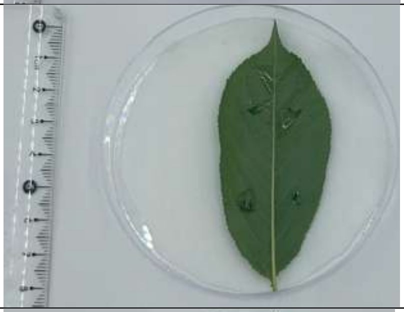
	apple “Aport Alexandr”	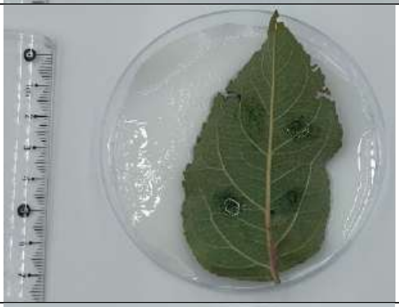
	apple “Burkhardt’s Renet”	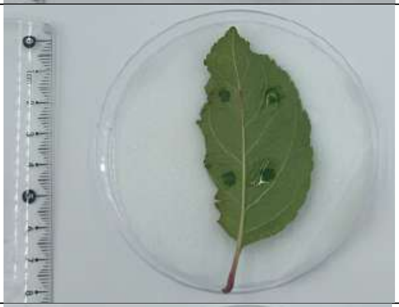
	apple “Red Delicious”	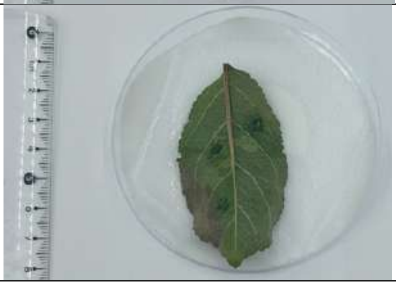

Ps02KZ	plum “Stanley”	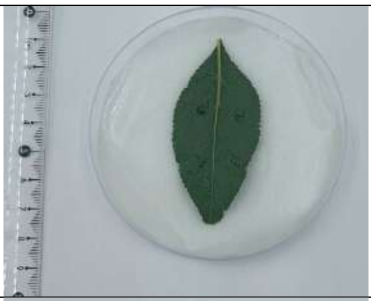	
	pear “Lesnaya Krasavitsa”	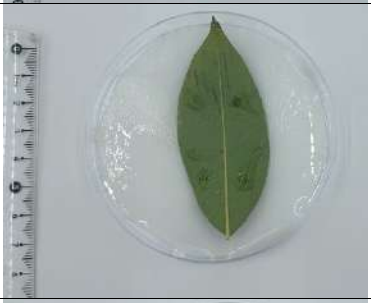	
	cherry “Lyubskaya”	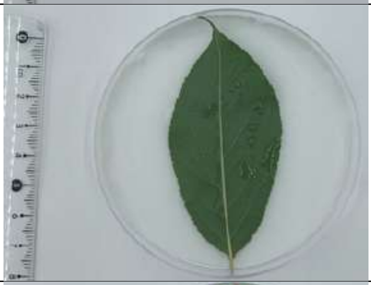	
	apple “Aport Alexandr”	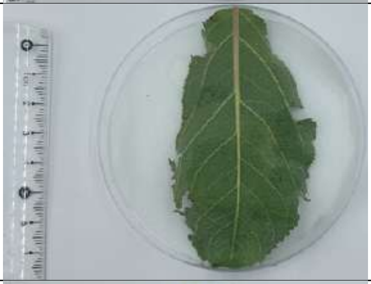	
	apple “Burkhardt’s Renet”	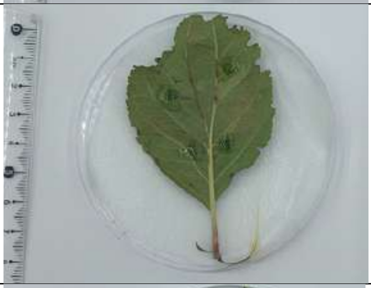	
	apple “Red Delicious”	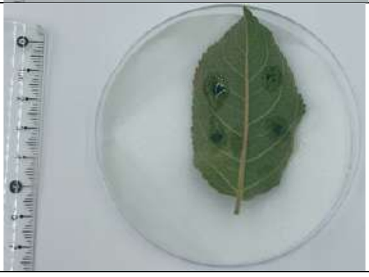	

Ps03KZ	plum “Stanley”	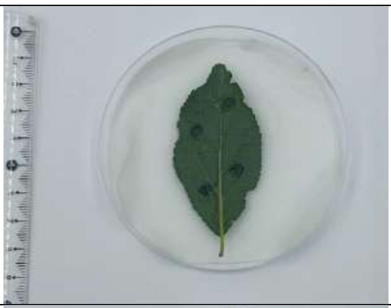	
	pear “Lesnaya Krasavitsa”	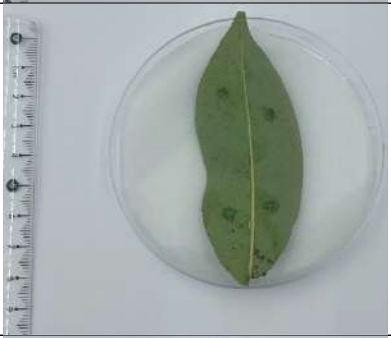	
	cherry “Lyubskaya”	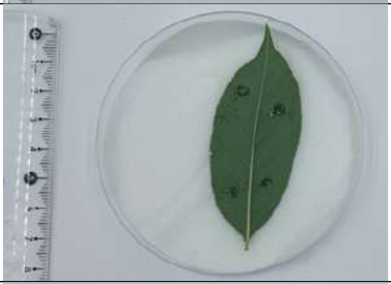	
	apple “Aport Alexandr”	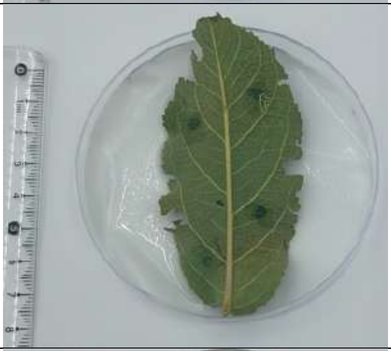	
	apple “Burkhardt’s Renet”	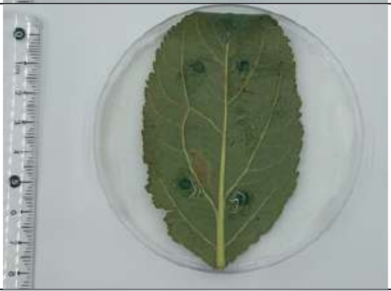	
	apple “Red Delicious”	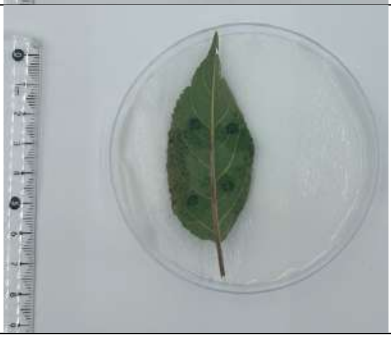	

Ea08KZ	plum "Stanley"	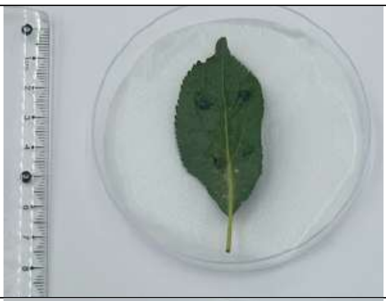	
	pear "Lesnaya Krasavitsa"	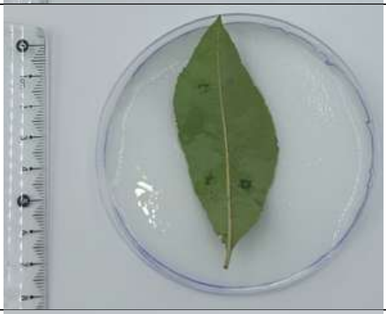	
	cherry "Lyubskaya"	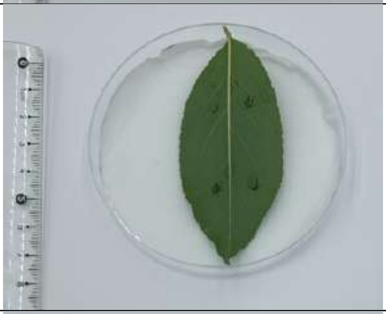	
	apple "Aport Alexandr"	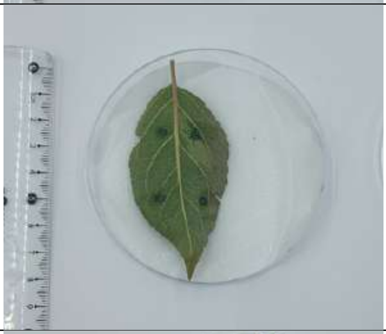	
	apple "Burkhardt's Renet"	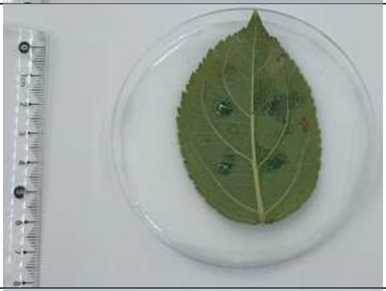	
	apple "Red Delicious"	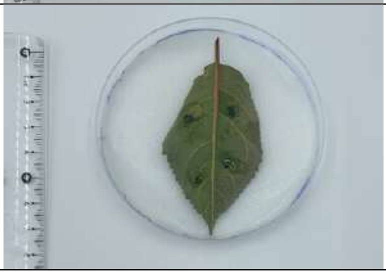	

Ea09KZ	plum "Stanley"	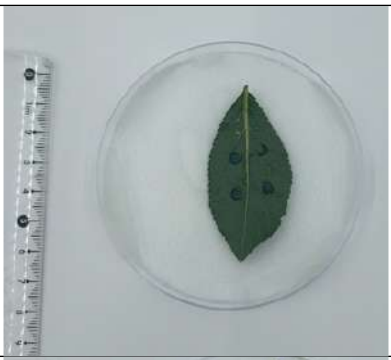	
	pear "Lesnaya Krasavitsa"	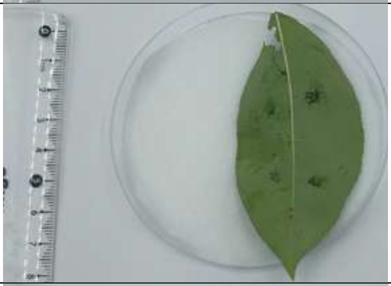	
	cherry "Lyubskaya"	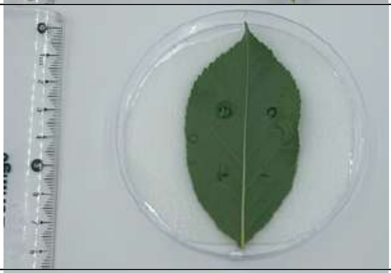	
	apple "Aport Alexandr"	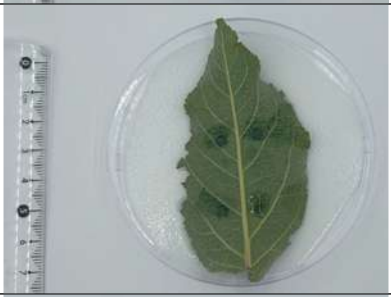	
	apple "Burkhardt's Renet"	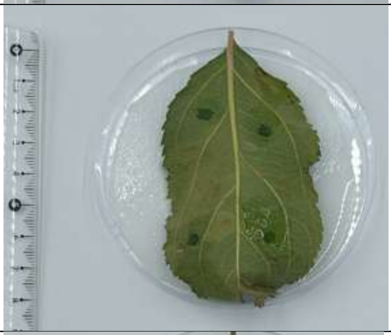	
	apple "Red Delicious"	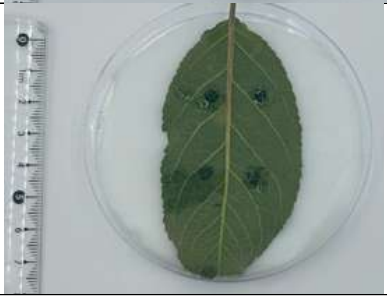	

K-	plum “Stanley”	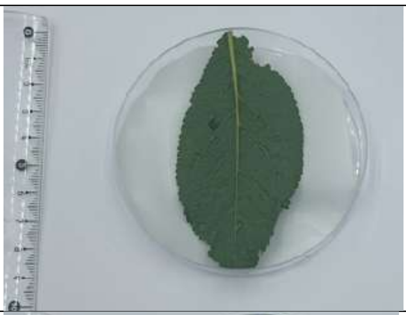
	pear “Lesnaya Krasavitsa”	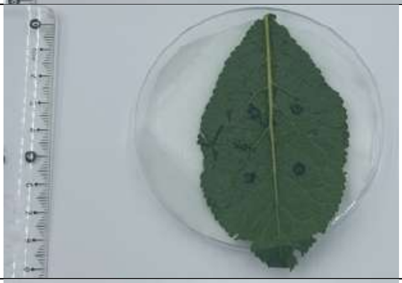
	cherry “Lyubskaya”	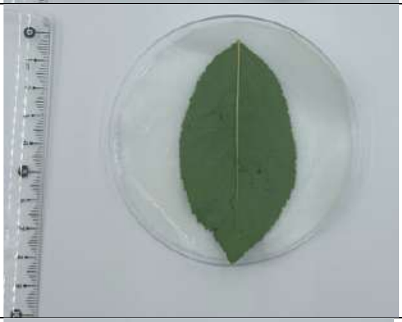
	apple “Aport Alexandr”	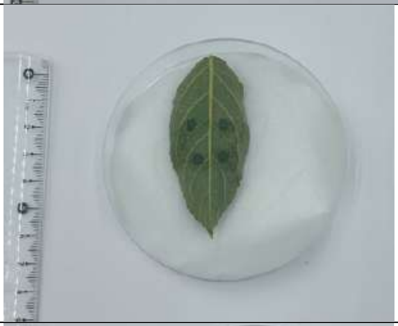
	apple “Burkhardt’s Renet”	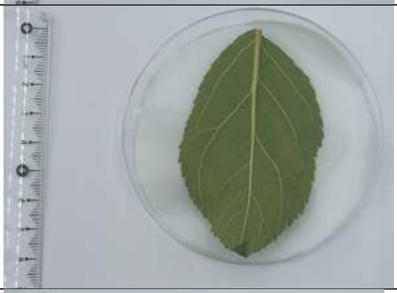
	apple “Red Delicious”	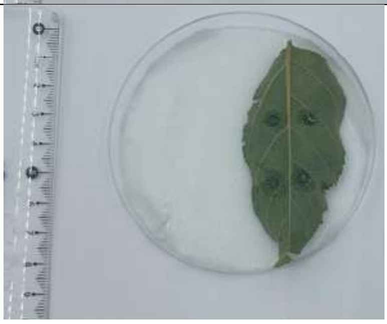

2 dpi		
Ps01KZ	plum “Stanley”	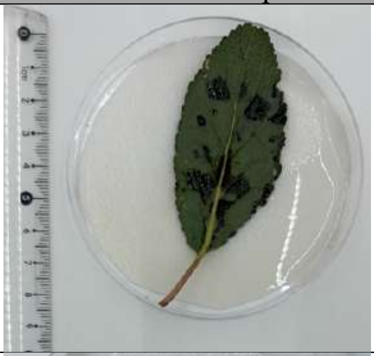
	pear “Lesnaya Krasavitsa”	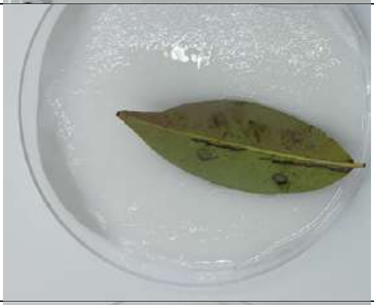
	cherry “Lyubskaya”	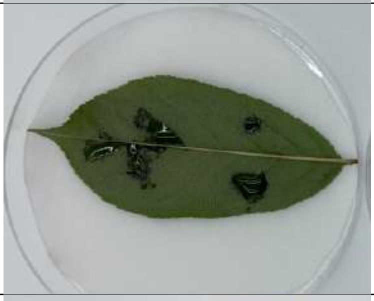
	apple “Aport Alexandr”	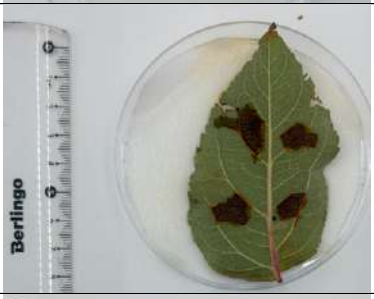
	apple “Burkhardt’s Renet”	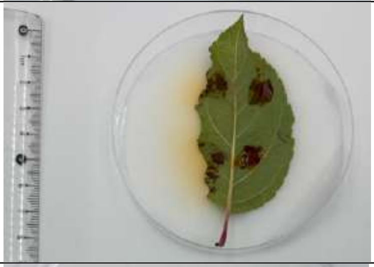
	apple “Red Delicious”	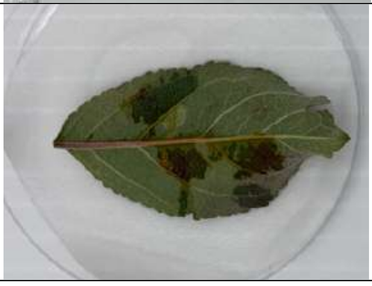

Ps02KZ	plum “Stanley”	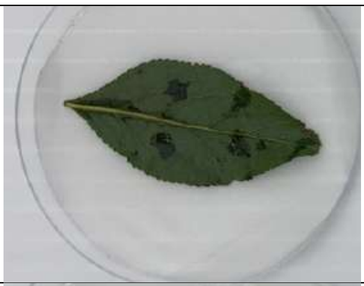
	pear “Lesnaya Krasavitsa”	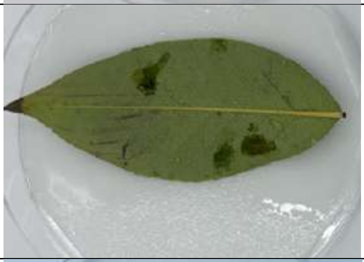
	cherry “Lyubskaya”	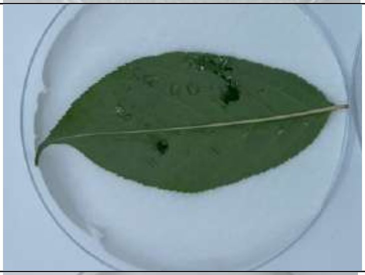
	apple “Aport Alexandr”	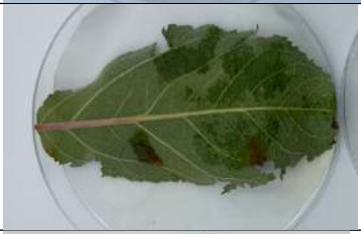
	apple “Burkhardt’s Renet”	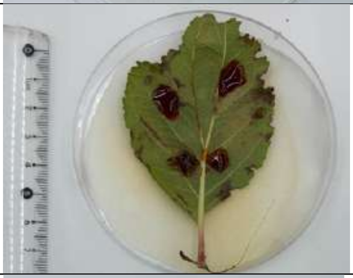
	apple “Red Delicious”	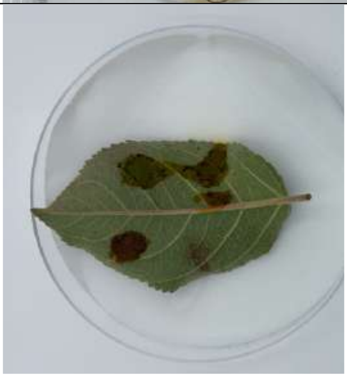

Ps03KZ	plum "Stanley"	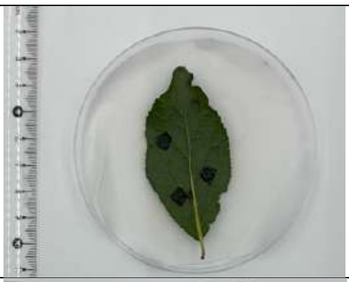
	pear "Lesnaya Krasavitsa"	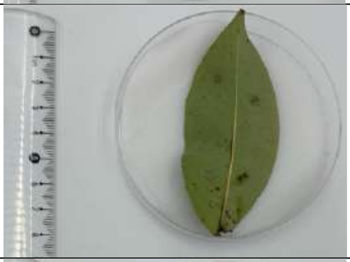
	cherry "Lyubskaya"	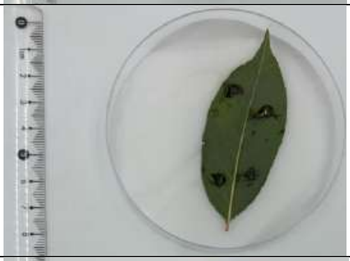
	apple "Aport Alexandr"	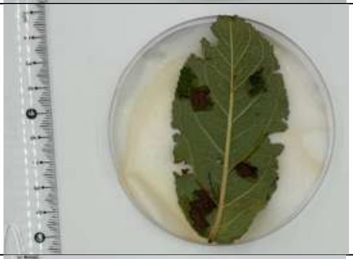
	apple "Burkhardt's Renet"	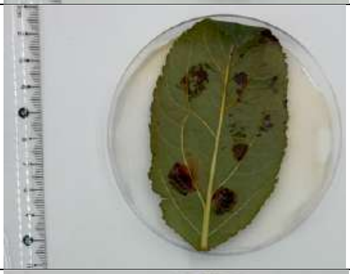
	apple "Red Delicious"	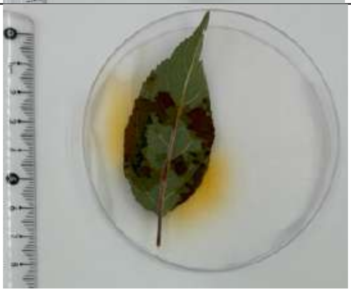

Ea08KZ	plum "Stanley"	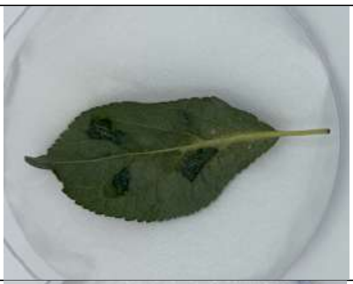
	pear "Lesnaya Krasavitsa"	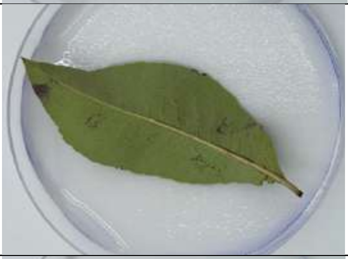
	cherry "Lyubskaya"	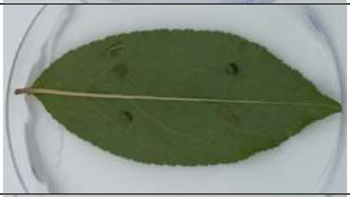
	apple "Aport Alexandr"	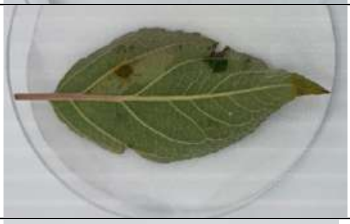
	apple "Burkhardt's Renet"	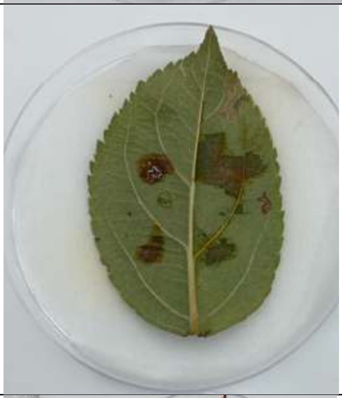
	apple "Red Delicious"	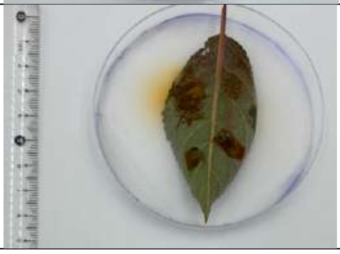

Ea09KZ	plum "Stanley"	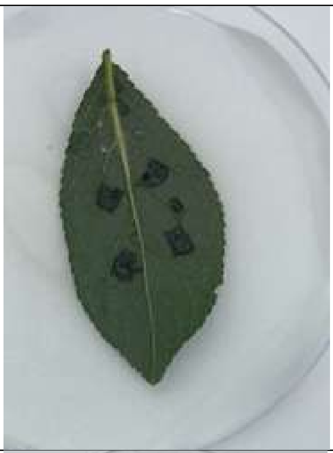
	pear "Lesnaya Krasavitsa"	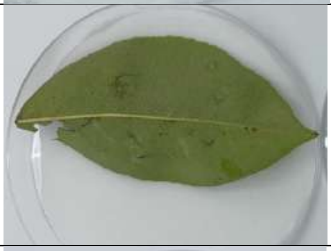
	cherry "Lyubskaya"	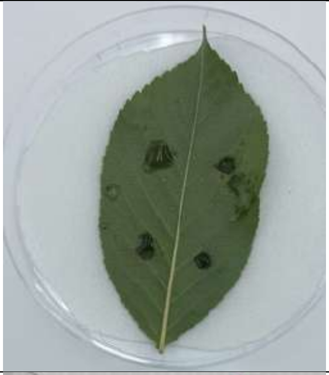
	apple "Aport Alexandr"	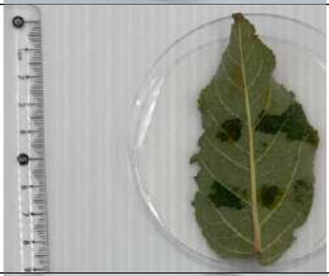
	apple "Burkhardt's Renet"	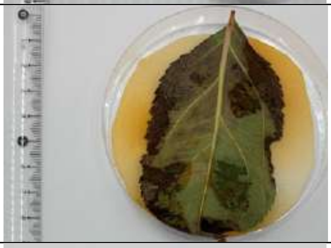
	apple "Red Delicious"	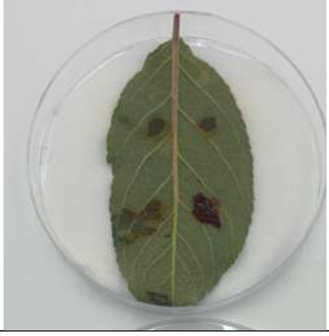

K-	plum “Stanley”	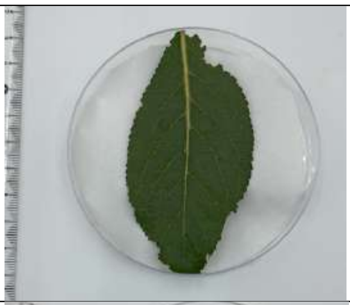
	pear “Lesnaya Krasavitsa”	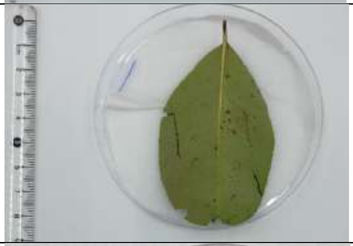
	cherry “Lyubskaya”	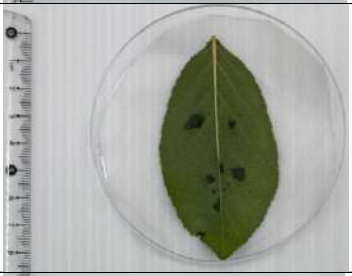
	apple “Aport Alexandr”	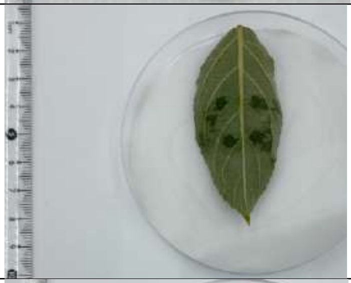
	apple “Burkhardt’s Renet”	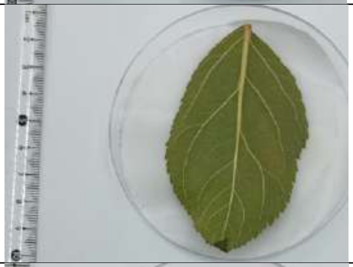
	apple “Red Delicious”	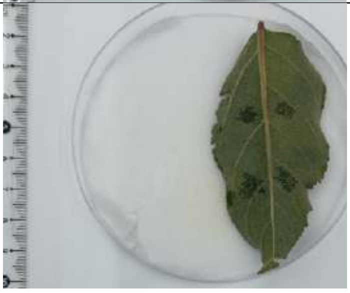

4 dpi		
Ps01KZ	plum "Stanley"	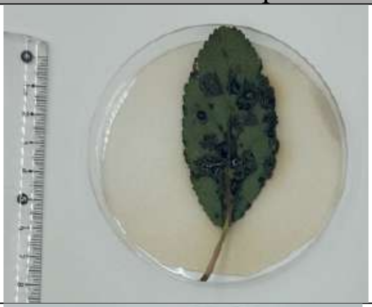
	pear "Lesnaya Krasavitsa"	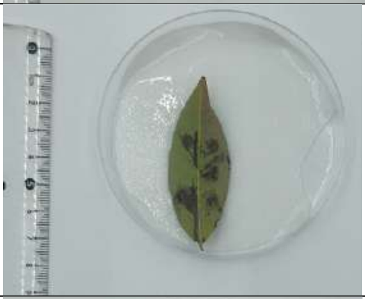
	cherry "Lyubskaya"	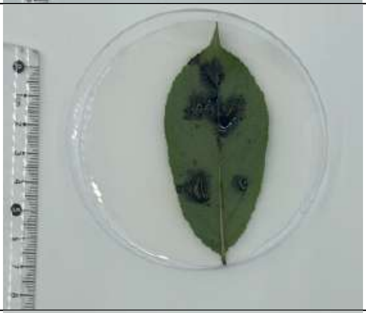
	apple "Aport Alexandr"	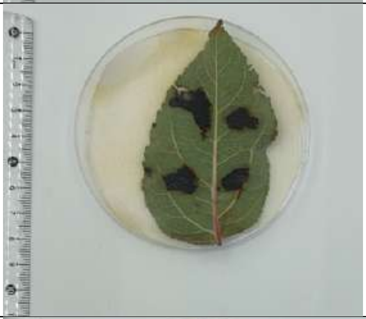
	apple "Burkhardt's Renet"	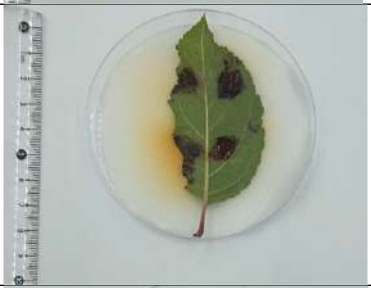
	apple "Red Delicious"	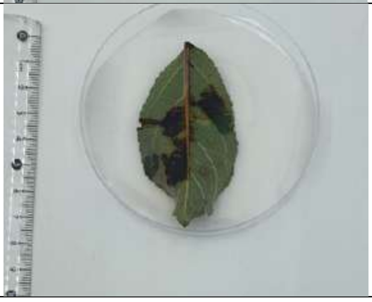

Ps02KZ	plum “Stanley”	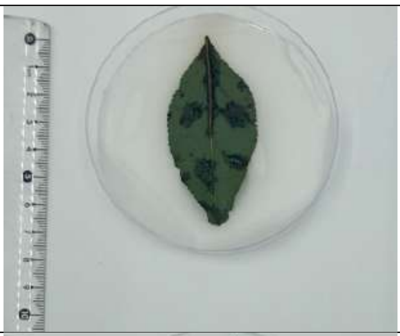
	pear “Lesnaya Krasavitsa”	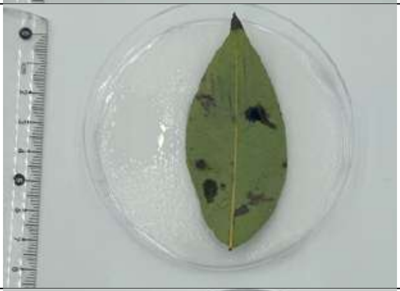
	cherry “Lyubskaya”	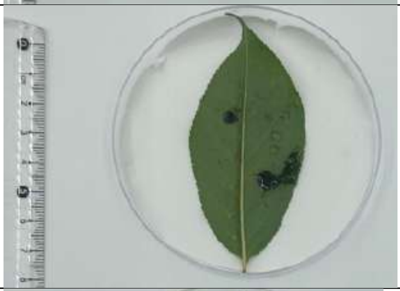
	apple “Aport Alexandr”	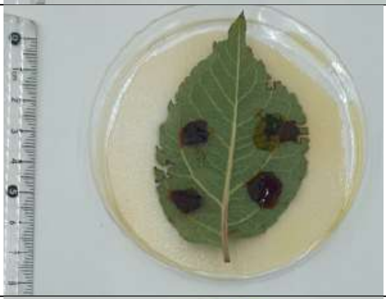
	apple “Burkhardt’s Renet”	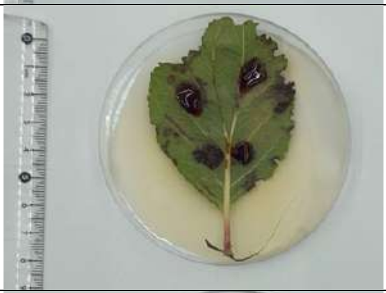
	apple “Red Delicious”	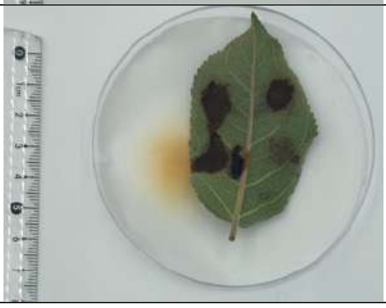

Ps03KZ	plum "Stanley"	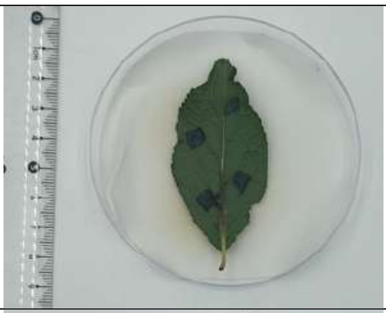	
	pear "Lesnaya Krasavitsa"	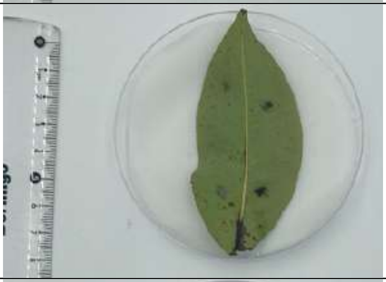	
	cherry "Lyubskaya"	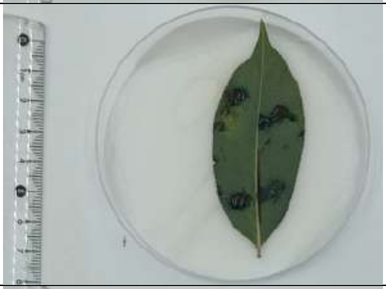	
	apple "Aport Alexandr"	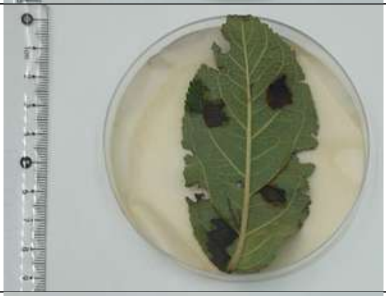	
	apple "Burkhardt's Renet"	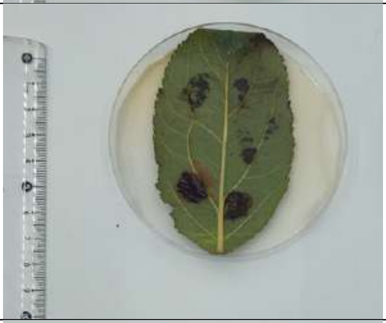	
	apple "Red Delicious"	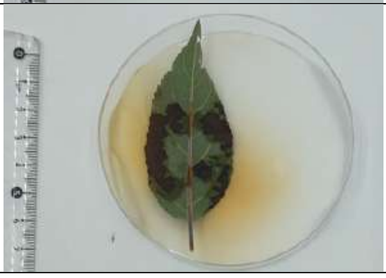	

Ea08KZ	plum “Stanley”	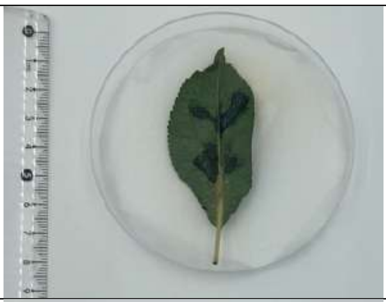
	pear “Lesnaya Krasavitsa”	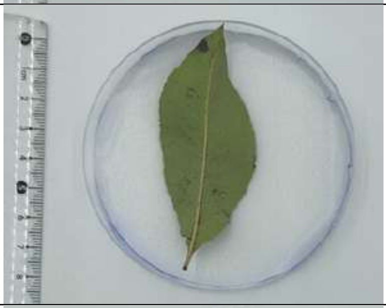
	cherry “Lyubskaya”	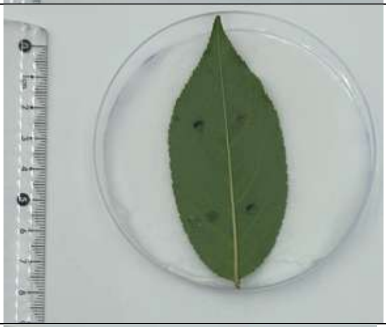
	apple “Aport Alexandr”	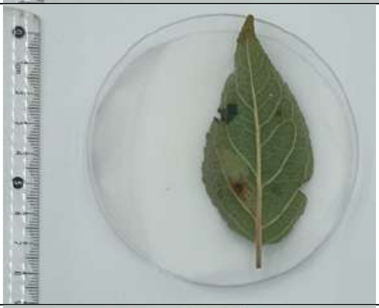
	apple “Burkhardt’s Renet”	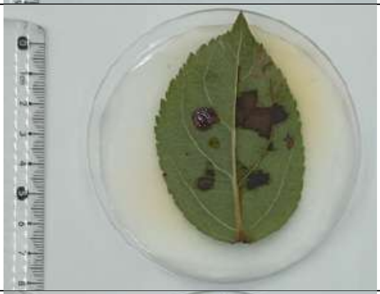
	apple “Red Delicious”	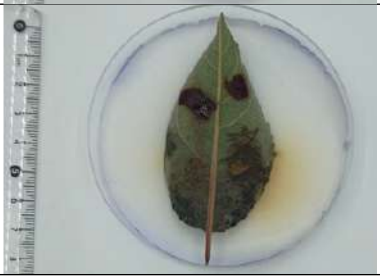

Ea09KZ	plum "Stanley"	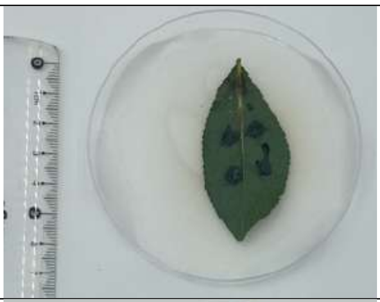
	pear "Lesnaya Krasavitsa"	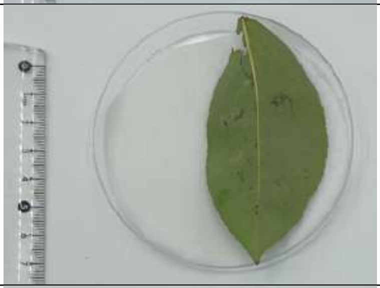
	cherry "Lyubskaya"	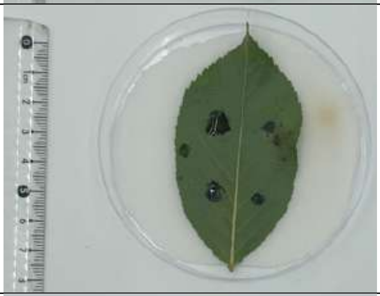
	apple "Aport Alexandr"	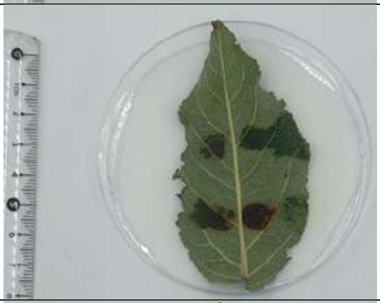
	apple "Burkhardt's Renet"	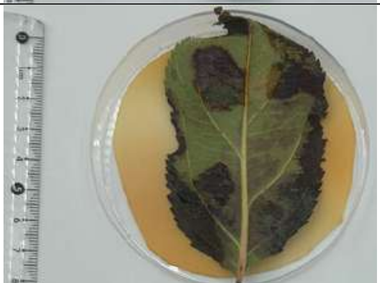
	apple "Red Delicious"	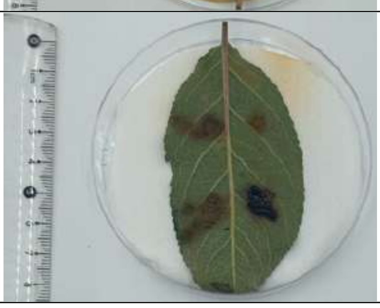

K-	plum “Stanley”	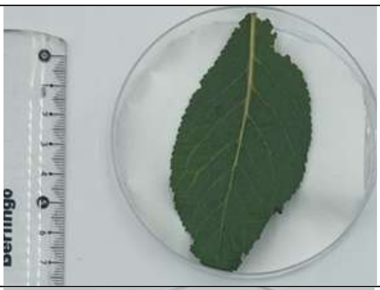	
	pear “Lesnaya Krasavitsa”	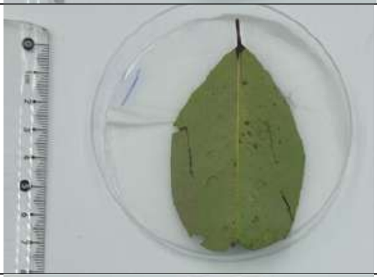	
	cherry “Lyubskaya”	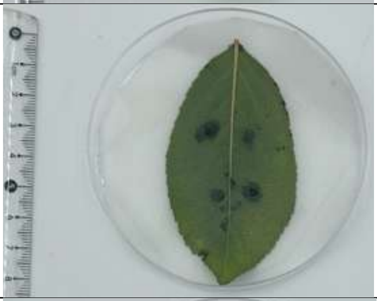	
	apple “Aport Alexandr”	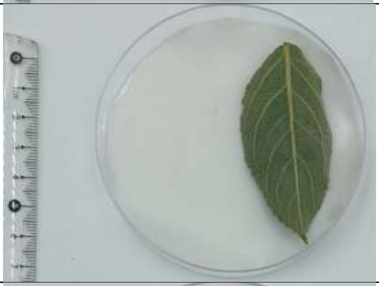	
	apple “Burkhardt’s Renet”	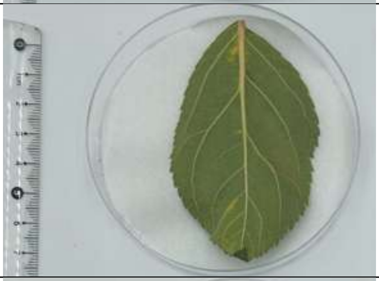	
	apple “Red Delicious”	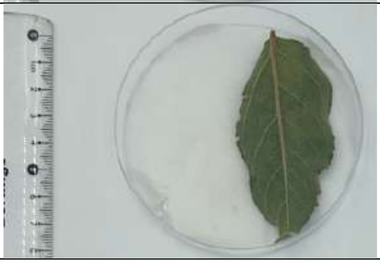	
